# Why don’t adolescent girls in a rural Uganda district initiate or complete routine 2-dose HPV vaccine series: Perspectives of adolescent girls, their caregivers, healthcare workers, community health workers and teachers

**DOI:** 10.1371/journal.pone.0253735

**Published:** 2021-06-29

**Authors:** Joseph Rujumba, Mathias Akugizibwe, Nicole E. Basta, Cecily Banura

**Affiliations:** 1 Department of Pediatrics and Child Health, Makerere University, Kampala, Uganda; 2 Kyambogo University, Kampala, Uganda; 3 Department of Epidemiology and Biostatistics, McGill University, Montreal, Canada; 4 Child Health and Development Centre, Makerere University, Kampala, Uganda; Zurich University, SWITZERLAND

## Abstract

**Introduction:**

Vaccination with the 2-dose HPV vaccine series among adolescent girls in Uganda remains low after almost 5 years since the vaccine was included into the routine national immunization program and barriers are not well understood.

**Objective:**

We explored barriers that prevent eligible girls from initiating or completing the recommended 2-dose HPV vaccine series in Oyam District, Northern Uganda.

**Methods:**

A qualitative study was conducted in Oyam District, Northern Uganda. Forty interviews were conducted with adolescent girls, their caregivers, Village Health Team Members, health workers and school administrators involved in HPV vaccination. All interviews were audio recorded and transcribed. NVivo version 11 was used for data management and content thematic approach for analysis guided by the Social Ecological Model.

**Results:**

At individual level, low levels of knowledge about the vaccine, girls’ frequent mobility between vaccine doses, school absenteeism and drop out, fear of injection pain and discouragement from caregivers or peers were key barriers. At the health facilities level, reported barriers included: few healthcare workers, inadequate knowledge about HPV vaccine, limited social mobilization and community engagement to promote the vaccine, limited availability of the HPV vaccine, unreliable transportation, lack of reminder strategies after the first dose of the vaccine, lack of vaccination strategy for out-of-school girls and un-friendly behaviour of some healthcare workers. Concerns about safety and efficacy of the vaccine, negative religious and cultural beliefs against vaccination, rumors and misconceptions about the vaccine, mistrust in government intentions to introduce the new vaccine targeting girls, busy schedules and the gendered nature of care work were key community level barriers.

**Conclusion:**

Our study revealed an interplay of barriers at individual, health facility and community levels, which prevent initiation and completion of HPV vaccination among adolescent girls. Strengthening HIV vaccination programs and ensuring high uptake requires providing appropriate information to the girls plus the community, school and health facility stakeholders; addressing cold chain challenges as well as adequate training of vaccinators to enable them respond to rumors about HPV vaccination.

## Introduction

Human papillomavirus [1] is an essential factor in the development of cervical cancer (CC). In 2018, an estimated 570,000 new cervical cancer cases and 311,000 deaths occurred in the world [2]. In 2018, the annual number of cervical cancer cases in Uganda were estimated at 6,413, with 4,301 annual cancer deaths [3]. Uganda has one of the highest annual CC rates in the world with age-standardized incidence rate of 54.8 per 100,000 and cancer-related deaths of 40.5 per 100,000 [3, 4]. Given the limited access to screening and early treatment of pre-cancerous lesions, prevention of HPV infection through vaccination could be the most critical intervention in the prevention of CC. Effective HPV vaccines targeting genotypes 16 & 18, which cause approximately 70% of CC cases worldwide are available in Uganda [5]. The World Health Organization recommends 2-dose HPV vaccine schedule for girls less than 15 years of age, with an interval of 6 months or as a 3-dose schedule for girls aged 15 years and above for maximum protection against HPV [6].

Uganda is one of the earliest Sub-Saharan African countries to implement an HPV vaccination demonstration project, which explored the feasibility of two vaccine delivery strategies; school grade versus age-based [7, 8]. At the conclusion of the demonstration project, the Ministry of Health [9] adopted a hybrid vaccine delivery strategy using both grade 4 girls and age-based (10-year old out-of-school girls) for scale up. Based on the WHO recommendations, the Ministry of Health (MoH) launched the 2-dose HPV vaccine series integrated in the routine Uganda National Expanded Program on Immunization (UNEPI) in November 2015 [8]. Currently, only 10-year-old girls both in- and out- of- school are targeted for vaccination.

In spite of the high HPV vaccine uptake during the demonstration project of 88.9% in a school based program [10], reported completion of routine 2-dose HPV vaccine series has so far been dismal. While the national initiation of the vaccine series was high [89%] in 2016, completion of the series was only 22% [9]. Of the 85% of eligible girls who initiated the HPV vaccine series in 2017, less than half (41%) completed the 2-dose series [11]. Similarly, a recent population-based cross-sectional study conducted among 460 girls aged 12–17 years found that almost half (49.6%) of the respondents did not initiate the vaccine series at all and only 18% completed the vaccine series [12]. Another population-based cross-sectional survey conducted among 407 girls aged 9–15 years found that 51% of the respondents were not vaccinated at all and only 14% had completed the vaccine series [13]. The findings from these population-based surveys imply that a significant proportion of adolescents do not get the full benefits of the HPV vaccine. While the long term vaccine efficacy of a single dose is still under investigation, incomplete dosing with the then recommended 3-dose schedule was inferior in the protection against cervical dysplasia and genital warts [14, 15]. Thus, both failure to initiate and failure to complete the HPV vaccine series is of particular public health concern. Since the HPV vaccine is a relatively new and recently integrated in the routine UNEPI [8], our objective was to explore the barriers to HPV vaccination among eligible adolescent girls in Oyam District, Northern Uganda.

## Materials and methods

### Study design and site

We conducted a study using qualitative methods of data collection between May and June 2018. Our study site was Oyam district, which is approximately 325 kilometers North of Kampala city. The district was selected purposively; being one of the 12 districts in which the HPV vaccination program was introduced as pilot in 2012 (3 years prior to integration of HPV vaccine into routine EPI). In 2014, the district had an estimated population of 383,644 people (187,121 males and 196,523 females). Majority (78.1%; 298,122) of the population is aged 30 years and below [16]. There were an estimated 63,000 children 6–12 years attending primary school and about half were females. The district is predominantly rural and comprises two counties also doubling as health sub-districts (HSD; North and South).

The 2 counties were divided administratively into 11 sub—counties and a Town Council covering a total area of about 2,716.2 square kilometers at the time of the study. The major economic activities included small scale subsistence farming and trade. The district was served with 235 government-aided and a few private primary schools. There were 29 government-owned and a few private health facilities at different levels within the 2 HSDs. While about 90% of households lived less than 5kms to the nearest primary school, only about one-third (33.1%) of households lived within 5 km to the nearest health facility [16].

### Study participants identification and selection

Participants included primary school girls and their caregivers, healthcare workers, Village Health Team members (VHTs) and teachers or school administrators.

To identify participants, we first purposively selected 4 health facilities from each HSD. Then, at each health facility, we asked the health center in-charge or manager to identify a healthcare worker who was involved in the HPV vaccination program activities as either a vaccinator or coordinator or supervisor. In turn, we asked the identified healthcare worker to identify a VHT who was either a vaccinator or mobilizer for HPV vaccination program. The VHT was asked to identify a girl who was eligible for vaccination from their community based on the inclusion criteria of the study, which specified the girl’s age and her vaccination status. The identified girl led the VHT to their adult caregiver. Subsequently, the healthcare worker was asked to identify either a teacher or school administrator who was their contact person for the HPV vaccination program at their respective school. Through this process, we compiled a list of potential study participants. The research assistants contacted the identified adult individuals and made appointments for further screening for eligibility. Whenever an identified individual was not eligible or was not interested in participating in the study, the process of identification and screening for eligibility for a replacement was repeated all over again. To conclude the identification process, eligible individuals who expressed interest in participating in the study were purposively selected based on inclusion criteria and participated in the informed consent process.

### Data collection procedure

We collected data using In-depth interviews (IDIs) and Key informant interviews (KIIs) as summarized in Table 1.

**Table 1:** Study population, type and number of interviews.

Study population	Data collection method	Sample size
Girl	IDI	8
Caregiver	KII	8
Healthcare Worker	KII	8
VHT	KII	8
Teacher or School Administrator	KII	8
**Total**		**8 IDIs and 32 KIIs**

### Development of interview guides and pre-testing

We developed both the IDI and KIIs guides using published literature compiled by a member of the study team into a question bank about HPV vaccination. We also brainstormed additional questions. We grouped the interview questions under broad themes and were translated in Langi presented in the guide as sub-texts to the English version of the questions. The guides were pre-tested among individuals with similar characteristics to potential participants according to the study inclusion criteria to ensure that questions were asked accurately and the meaning understood.

### In-depth interviews (IDIs)

Using the guide, we conducted IDIs with the girls in Langi at venues selected by their caregivers. The interview questions explored the girls’ knowledge about the HPV vaccine and vaccination as a preventive intervention against cervical cancer; their perceptions about the HPV vaccine and vaccination experiences as well as their decision making about taking the vaccine or not.

### Key informant interviews (KIIs)

We used the same guide to conduct interviews with caregivers, healthcare workers, VHTs and teachers or school administrators. Some KIIs questions were tailored to the category of participant. Interviews with all participants explored their knowledge or awareness about the HPV vaccine, vaccination service delivery, and community perceptions about the HPV vaccine and vaccination. Caregivers and teachers were also asked questions on awareness of vaccination activities. Interviews with healthcare workers and VHTs also explored their knowledge about national vaccination policy, capacity of health facility workforce to deliver the vaccine to recipients, and availability of vaccines, cold chain infrastructure and logistics. Caregivers preferred to be interviewed using Langi and the other participants preferred the English language.

For practical reasons, different research assistants conducted interviews with girls and their caregivers on the same day but independent of each other. Interviews with the girls lasted on average 30 minutes. Interviews with caregivers, healthcare workers, VHTs and teachers or school administrators were scheduled at the convenience of the participant and each lasted about 45–60 minutes. All interviews were audio- recorded with permission from the participant.

### Data management and analysis

Two research assistants fluent in both Langi and English and with experience in conducting interviews were recruited and trained on the objectives of the study, eligibility criteria for participant selection, the process of data collection and management. The research assistants provided daily progress reports to the principal and co-principal investigators on data collection and emerging issues. All audio-recorded interviews were transcribed at the end of data collection. All transcribed interviews were checked against the audio-taped versions for accuracy and completeness. Half of the Langi transcribed interviews were checked by an independent research assistant and half of the English transcribed interviews were checked by the co-PI. The Langi transcribed scripts were subsequently translated into English. We used the broad themes of the interview guides for the first level of analysis. Sub-themes emerging within each broad theme were identified and these formed the basis for the construction of a code book. The first and second authors met to discuss and harmonize the code book. The draft codes and preliminary findings from this phase were discussed with co-authors in a de-briefing meeting.

The English transcripts were exported to and coded using QSR International’s NVivo version 11 and analyzed using a content thematic approach [17]. Drawing on the Social Ecological Model (SEM) [18–21] as an analytical framework, sub-themes emerging from the data were grouped under SEM themes for interpretation. SEM was selected to provide a basis for identifying and locating barriers at individual, organizational (health facility) and community levels. Quotations illustrative of the barriers to vaccination were identified and used in the presentation of the study findings. We conducted concurrent triangulation, which involved the analysis of findings from the girls, their caregivers as well as those from healthcare workers, VHTs and teachers or school administrators. Throughout the process of data processing and interpretation, identities of participants were masked to maintain anonymity.

### Ethical considerations

The study was approved by Makerere University School of Medicine Research and Ethics Committee (SOMREC), Uganda National Council for Science and Technology (UNCST), and the University of Minnesota Institutional Review Board. Adult study participants provided written informed consent and minors provided verbal assent after study staff obtained written informed consent from their caregivers.

## Results

Participant categories and age are summarized in Table 2.

**Table 2:** Participant category and age.

Participant Category	Average Age (range) years
Adolescent girls*	12.0 (10–15)
Caregivers	39.3 (25–56)
Healthcare Workers**	39.3 (26–57)
VHTs***	40.4 (32–47)
Teacher/school administrator****	36.0 (26–45)

*3 girls received 2 doses (fully vaccinated), 3 girls received only 1 dose (incomplete vaccination), 2 unvaccinated girls.

** 4 Nursing assistants & 4 Nurse and/or midwife.

*** VHT- 6 vaccinators and 2 mobilizers.

**** 7 teachers & 1 head teacher (school administrator).

### Barriers to HPV vaccination

Barriers to HPV vaccination were grouped under the three SEM themes: i) individual ii) health facility and iii) community. Sub-themes under each of the themes are summarized in Table 3.

**Table 3:** Thematic presentation of barriers to HPV vaccination in Oyam District.

Major Theme	Sub-theme–barrier
**Individual**	- Inadequate knowledge about the HPV vaccine - Caregivers’ lack of awareness of vaccine and vaccination activities - Change of residential location or school within or outside the district between doses - Absenteeism or dropout from school - Fear of injection pain - Discouragement from vaccination by caregivers or peers
**Health facility**	- Limited healthcare workers’, VHTs’ and teachers’ knowledge about HPV vaccine and national HPV vaccination policy - Lack of strategies targeting out-of-school girls - Vaccine supply shortages and inadequacies of cold chain infrastructure - Limited social mobilization and community engagement activities - Unpredictable transportation for staff and vaccine resulting in irregular vaccination outreach events to schools and communities - Unfulfilled healthcare staff expectation to be paid extra allowances and financial incentives for teachers - Few healthcare workers at health facilities to deliver the vaccine to recipients - Un-friendly healthcare workers - Lack of reminder/recall strategies for 2nd vaccine dose
**Community**	- Rumors and misconceptions about the vaccine and vaccination - Strong traditional and religious beliefs - Mistrust of government intention of introducing new vaccines - Busy schedules and gendered nature of domestic work

#### (i) Individual level barriers

*Inadequate knowledge about the HPV vaccine*. The girls had inadequate knowledge about the vaccine. They were not sure about the name of the vaccine they received, the infection/disease the vaccine protected them against and the number of doses they were supposed to receive ‘*They (healthcare workers) said that it [the vaccine protects against] is Candida*, *AIDs*, *elephantiasis’ (Girl Health Facility # 6); ‘*T*hey (girls) are to be vaccinated twice or three times there is no problem with that’ (Girl*, *Health facility# 3)*. *‘*The girls’ inadequate knowledge about the vaccine was corroborated by healthcare workers. ‘*Most of them (girls) thought it (the vaccine) was Tetanus Toxoid’ (HCW Health facility# 2)*.

*Caregivers’ lack of awareness of the HPV vaccine and vaccination activities*. Caregivers reported lack of information about the vaccine and inadequate information about vaccination program activities. Some caregivers reported learning about the vaccination from their daughters after vaccination at school. Caregivers also reported that their daughters did not know why they were vaccinated and the disease/infection the vaccine was meant to prevent. ‘*She only told me that she went to school and she got vaccinated but did not tell me the reason why and what disease they were vaccinated against’ (Caregiver*, *Health Facility# 4)*. Caregivers did not know who was eligible for vaccination and therefore were not in a position to provide informed guidance to their daughters. ‘*They vaccinate girls who are 10 years and below’ (Caregiver*, *Health facility#7)*. Healthcare workers confirmed caregivers’ lack of information about the vaccine and vaccination program activities. ‘*They are lacking knowledge on what HPV vaccine is’ (HCW Health facility #8)*. *‘Parents are not informed…’ (HCW*, *Health facility#5)*.

*Change of the girls’ residential location or school within or outside the district between doses*. Healthcare workers reported that change in residential location or school within or outside the district after initiation of the vaccine series resulted in non-completion for many girls. ‘*We had given them first dose we went to give them the second dose but they were nowhere to be seen*. *We tried tracing them but their parents’ had transferred them to another school in another district’ (HCW*, *health facility#2)*. Some of the girls whose relatives changed residential location particularly those who moved out of the district would miss vaccination altogether. *‘*Y*ou find that the mother will relocate with the girls so they will not get the vaccine’ (VHT*, *Health facility# 6)*. VHT information was corroborated by the teachers. ‘*Some girls stay with their relatives and get the first dose but the second dose comes when they have gone to their parents so they miss the second dose’ (Teacher*, *School within the catchment area of health facility# 6)*.

*Absenteeism and drop out from school*. Healthcare workers and girls reported absenteeism from school on vaccination day as a barrier to vaccination. Reasons for absenteeism were varied but included menstruation, girls sent back home because of unpaid school tuition or other fees, girls asked to skip school by their caregivers. ‘*You know some of the p4 girls have already started menses and the girls when they are in their menses they don’t attend school that day they stay home’ (HCW*, *Health facility#7)*. *‘Some (girls) have been sent home for issues of school fees…’ (HCW*, *Health facility#5)*. *‘If they have not paid either they are sent to go pick the PTA fees and you find that when it happens to be the day of vaccination then the girl will not be vaccinated’ (HCW*, *Health facility#7)*.

At times, absenteeism was due to caregivers stopping the girls from attending school to help out with domestic work or gardening. ‘*There was a lot of work at home’ (Girl*, *Health facility 4)*. *‘You find that the day the vaccinators are going to the school*, *the parent could have stopped the child from going to school because of too much work in the garden’ (VHT*, *Health facility #1)*. Healthcare workers also reported that sometimes lack of coordination between the school and health facility in scheduling of vaccination sessions resulted in missed opportunities to vaccinate the girls. ‘…*we missed out some girls because we went in April during exam time’ (HCW*, *Health Facility#5)*. *‘When they (healthcare workers) have the program (3) in school ah*! *they should send the information early*, *but here what they do*, *you just see them coming with their packages and we want this kind of girls here so that if they would send information in advance*, *it would be fair to help in mobilization so that those who are at home come (for vaccination)’ (Teacher*, *School in catchment area of Health facility# 2)*.

Girls’ dropping out of school between doses to get married or because they became pregnant coupled with fear and embarrassment to return to their former schools to complete the vaccine series was a challenge to the healthcare workers, VHTs, caregivers and teachers. ‘*Some of them (girls) they get the first dose like the ones who are in P*.*4 (age range 9–15+ years) most of them get married before the second dose and they disappear’ (HCW*, *Health facility# 3)*. *‘You can give the girl the first vaccine you will come back and find that the girl is already married or pregnant and when she is pregnant she will feel embarrassed to come back for the vaccine’ (VHT*, *Health facility#3)*. *‘Other girls after dropping out of school*, *they now fear going back to school (for vaccination)’ (Caregiver*, *Health facility #8)*. Teachers and healthcare workers reported being unable to reach the girls who dropped out of school with the missed vaccine dose. *‘They are in the villages and there is no way we can connect with them to come for the second dose of the vaccine’ (Teacher*, *School in catchment area of Health facility# 6)*. *‘You find that they (girls) have left school or transferred to another school so we cannot go to trace them from their homes since we do not know their homes’ (HCW*, *Health facility# 4)*.

Physical barriers such as impassable roads during the rainy season prevented girls and vaccinators from reaching vaccination venues. *‘One thing is that some girls are in the hard-to-reach areas and you find that with changing weather it is hard for them to reach the facility or the facility to reach them because the roads to some outreaches are impassable during the rainy season’ (HCW*, *Health facility# 5)*. One of the teachers admitted to girls’ not being given information about alternative venues for vaccination if they missed getting their dose at school. ‘*I have never heard them mobilizing people to come for these vaccines in the health centers no*, *I can’t lie’ (Teacher*, *School in catchment area of Health facility# 2)*.

*Fear of injection pain*. All participants reported some girls refusing the second vaccine dose and some of them being influenced by their peers not to take the vaccine on grounds that the injection is painful. ‘*They (girls) feared injection because it was said that the injection is painful’ (Girl*, *Health facility#4)*. *‘Most of the girls fear injections’ (Teacher*, *School in catchment area of Health facility#5)*. *‘When you go to vaccinate them (girls) they fear injection and dodge being vaccinated’ (VHT*, *Health facility#1)*. *‘I heard some of the girls say that this injection is very painful and she was saying that she will not go back for the second dose’ (VHT*, *Health facility#3)*. *‘Some of the girls also go for vaccination and when they hear their friends saying that the needle is painful*, *they will pretend that they too have been vaccinated when they are not’ (VHT*, *Health facility #2)*.

*Discouragement from vaccination by caregivers or peers*. Teachers and VHTs reported caregivers who were against any form of vaccination and therefore were not in favor of their daughters’ vaccination at school. ‘*Like in our school here*, *there are girls who have totally not been vaccinated*. *When times come they run away that their parents said that they should not be vaccinated’ (Teacher*, *school in catchment area of Health facility# 4)*. *‘Some of them may go back home and tell their parents that they were vaccinated then the parents will tell her that she should not go back that the vaccine is bad they should not go back for it then you will find some girls are not coming back for the vaccine’ (VHT*, *Health facility#7)*.

While parental consent was not required prior to vaccination, some girls used it as an excuse to refuse vaccination and caregivers used the opportunity to discourage their daughters from vaccination. ‘*Some of the girls want to consult with their parents and get parental consent before vaccination*. *In some cases*, *girls are discouraged by their parents and others use it as an excuse to dodge the vaccination’ (Teacher*, *School in catchment area of Health facility#2)*. Teachers also reported girls being discouraged by their peers particularly for the second dose, which they attributed to inadequate knowledge about the benefits of completing the vaccine series. ‘*There are some peers who even can tell them …you now don’t go that one is enough now you have already got the vaccine why are you going for another one’ (Teacher*, *School in catchment areas of Health facility# 3)*.

*Girls’ reported refusal to be vaccinated*. Several VHTs noted that some girls refused to get vaccinated without giving any reason. Then, others refused vaccination because they did not consider it useful. ‘*Some of the girls just refuse to go for vaccination’ (VHT*, *Health facility# 5)*. *‘These girls are lazy even if you tell them to go for vaccination so they just do not go because of laziness and they do not take it (vaccination) seriously’ (VHT*, *Health facility#3)*. In some instances, the girls were reported to accept vaccination after the teachers’ intervention. *‘Like during the last one (Vaccination exercise) some of the girls were just forced by the teachers to come and get vaccinated*. *If they were not forced*, *they would not have come for the vaccine’ (VHT*, *Health facility#7)*.

#### (ii) Health facility barriers to HPV vaccination

*Limited healthcare workers’ VHTs’ and teachers’ knowledge about the HPV vaccine and the national HPV vaccination policy*. Healthcare workers and VHTs who are the frontline workers for the vaccination program had gaps in knowledge about the vaccine, side effects and the national HPV vaccination policy. Both healthcare workers and VHTs had incorrect knowledge about the vaccine. ‘*The vaccine is a live virus*. *It (3) is a vaccine to protect young and sexually active girls against the cancer of the cervix’ (HCW*, *Health facility# 3)*. Some healthcare workers and VHTs did not know the MOH recommended eligible target age-group for vaccination. ‘*We have to vaccinate all girls of 9 years at schools’ (HCW*, *Health facility# 8)*. *‘They should bring girls between the ages of 9 to 12 years who are not in school to come and be vaccinated*. *If the girl is in P4 and is 14 or 15 years they do not vaccinate them because they tell them that the vaccine is only for girls who are 10*, *11*, *and 12 years because older girls will already be sexually active’ (VHT*, *Health facility# 6)*. *‘They say that we should give girls who are 9*, *10 because above that past 10 years they are already sexually active’ (VHT*, *Health facility# 7)*. Both the VHTs and teachers acknowledged their inadequate knowledge about the vaccine. *‘We lack knowledge about HPV vaccine (we) they just know that we are going to vaccinate the girls but do not know what it is*’ (VHT, *Health facility#* 3). ‘*We know very little about the vaccine and think we should be given a manual that tells us more about the vaccine’ (Teacher*, *school in catchment area of Health facility# 6)*. *A*ccordingly, teachers recognized their limitation to provide accurate information about the vaccine to the girls. ‘*My view is that you don’t leave the vaccination exercise to us the teachers because we don’t know the criteria [for vaccination] and what to tell them (girls) at times we just say what is not supposed to be said’ (Teacher*, *school in catchment area of Health facility# 3)*. Since some of the teachers were not familiar with the national HPV vaccination policy, they wondered why the vaccine was only given to a few girls in school. ‘*The vaccine is given to just a few girls who are at school and in P*.*4 only*, *yet some of them in P*.*6 and P*.*7 are in the same age bracket’ (Teacher*, *school in catchment area of Health facility#6)*.

*Lack of strategies targeting out-of-school girls*. The teachers’ insufficient knowledge about the national HPV vaccination policy lead them to believe that vaccination only targeted school girls. There was lack of coherent plan to reach out -of -school girls including those who dropped out with information about the vaccine and vaccination program activities thus heightening their disadvantage to access the vaccine. ‘*If am to tell you most of the girls in the village are suffering because most times you can hear that they have vaccinated children from school but in the villages they have not got the vaccine’ (Teacher*, *school in catchment area of Health facility# 5)*. Another teacher said: ‘*Girls out of school they don’t get*. *We give only girls in schools’ (Teacher*, *school in catchment area of Health facility# 6)*.

*Vaccine supply shortages and inadequacies of the cold chain infrastructure*. All participants reported vaccine shortages as a barrier to vaccination, which they attributed to either inadequate doses or outright stock- outs. ‘W*hen you are supposed to go and vaccinate these 10-year old girls and find that the vaccine is out of stock and the quantity they bring is not enough’ (HCW*, *Health facility#1)*. ‘*You may go to get the vaccine and you don’t find the vaccine and yet these girls have been mobilized already they (the girls) are there waiting*, *you end up by going to the community and you tell them*, *sorry the HPV vaccine is out of stock may be next time*, *that is what demoralize them so much’ (HCW*, *Health facility# 1)*. *‘The vaccine got finished’ (Girl*, *health facility# 7)*. *‘She (health worker) told me that the vaccine got finished before my daughter was vaccinated…’ (Caregiver*, *Health facility# 7)*. According to VHTs, some girls were reported to get more doses than recommended by the MoH contributing to the vaccine shortages. ‘*Some of the girls just change school you find that she was vaccinated in this school but will transfer and start getting fresh vaccination in the new school’ (VHT*, *Health Facility#5)*. Both VHTs and teachers expressed frustration when mobilized girls were not vaccinated. Such missed opportunities discouraged the girls to return another time when the vaccine was available. ‘*Some of the girls were left out because the vaccine got finished*. *We even went up to the district and they said that it is not there and they said that if they bring then we will vaccinate the remaining girls’ (VHT*, *Health facility# 3)*. *‘With HPV vaccine we always have shortages of vaccine*. *The vaccine is not enough this is making some of the girls to miss the vaccine’ (HCW*, *Health facility# 6)*. However, there was no guarantee that girls who missed their vaccine dose on vaccination day would receive it when it became available. *‘At times they (girls) have the interest of getting that vaccine when they go to the center where the service is being provided you find that what is there is not enough for all of them’ (Teacher*, *School in catchment area of Health facility# 7)*.

Inadequacies of the cold chain infrastructure and other logistics were another challenge healthcare workers had to deal with in order to deliver the vaccine to the girls. For instance, some health facilities lacked or had unrepaired vaccine fridges. ‘*We do not have a fridge so if a person comes and does not get the service she will end up being discouraged from coming back but if we had the fridge we would be having the vaccine at all times’ (HCW*, *Health facility# 6)*. Additionally, healthcare workers reported inadequate vaccine carrier ice packs, which compel vaccinators to carry few vaccine doses. ‘*When we are going for vaccination*, *the logistics are not enough like the vaccine carrier ice packs it is not enough’ (HCW*, *Health facility# 1)*.

*Limited social mobilization and community engagement activities*. Healthcare workers reported limited community sensitization activities to raise awareness about the vaccine and vaccination activities. ‘*Most families were not aware*. *Healthcare workers would just vaccinate these girls without telling them why they were vaccinating them’ (HCW*, *Health facility# 2)*. As a result, caregivers were excluded from the vaccination program activities and sometimes had no clue about their daughters’ vaccination status. ‘*Actually*, *I did not know that my daughter did not complete her vaccination’ (Caregiver*, *Health facility# 3)*. According to healthcare workers, seasonal activities determined the girls’ access to vaccination information and services. *‘This season people are cultivating … they spend their time in the gardens when the mobilizer is passing around …they do not find them (girls & their caregivers) so they miss the information and they end up not being vaccinated’ (HCW*, *Health facility#1)*.

*Unpredictable transportation for staff and vaccine resulting in irregular outreaches in schools and communities*. Transportation of staff and vaccine was reported by VHTs as unpredictable and made planning for vaccination unfeasible. ‘*We only had a problem of transport for getting this vaccine from Oyam district headquarters’ (VHT*, *Health facility# 6*). ‘*Transport is another problem from here we normally use bicycles and at times you can get a bike to go outside to vaccinate the girls and the bike again get spoilt on the way leading to waste of time and you reach the vaccination point late’ (VHT*, *Health facility# 1)*. Consequently, scheduled vaccination in schools were either delayed or did not happen at all. ‘*Sometimes they (HCWs) say that there will be vaccination and the children wait even for three days in vain*. *In other cases*, *they (HWs) come like at 2 o’clock when the children have gone for lunch and some do not return to school’ (Caregiver*, *Health facility# 3)*. Long waiting time discouraged some girls as they would leave without getting vaccinated. *‘The healthcare workers should follow time when they are going for the vaccination they should not take so long before going and keeping these pupils waiting this will discourage them and they will go away’ (VHT*, *Health facility #8)*. Due to the unpredictable nature of scheduled vaccination sessions, some caregivers were reported to prevent their daughters from returning to vaccination sites. *‘Caregivers will not allow them (the girls) to come back for the second time since the day they came they stayed till the evening and went back minus being vaccinated now the parents will say no*, *they will not allow them to come’ (HCW*, *Health facility# 1)*.

*Unfulfilled healthcare workers’ expectation to be paid extra allowances and financial incentives for teachers*. Both the VHTs and teachers expected to receive some monetary allowance or incentive every time vaccination took place. ‘*Actually we need to be given something (allowance) for moving because sometimes others (VHTs) even have given up it’s not easy to move*. *When you are not motivated sometimes even you sit and relax but if there is something to be given you will be serious about it’ (VHT*, *Health facility# 4)*. *‘Vaccinators need to be motivated as well (VHT*, *Health facility# 1)*. Teachers, on the other hand, expected to be given a monetary incentive for mobilizing eligible school girls for vaccination. *‘You will go and find that the teachers are not interested in the activity (vaccination exercise) and they are so lazy and they think that they (healthcare workers) have come to make money so that too can make the girls fail (to be vaccinated)’ (HCW*, *Health facility# 6)*.

*Few healthcare workers at health facilities to deliver the vaccine to recipients*. VHTs reported that few available healthcare workers sometimes prevented the delivery of HPV vaccine to girls particularly at health facilities. ‘*We have only 3 people who go to vaccinate people i*.*e*. *the health assistant and us (VHTs) at times the in-charge also goes but we cannot leave the health facility without any one’ (VHT*, *Health facility# 6)*. ‘*When they (girls) come for vaccination and find when we (VHTs) are not there and find when the healthcare workers are very busy in other areas and they will not have time to vaccinate these girls’ (VHT*, *Health facility# 7)*. Consequently, girls reported not being vaccinated because the few available healthcare workers were busy elsewhere providing other health services. ‘I *said I will first go and see*, *people were many (at health facility) so I did not get vaccinated’ (Girl*, *Health facility# 6)*.

VHTs and teachers also reported unfriendly behavior exhibited by some healthcare workers, which frightened some of the girls. ‘*Healthcare workers should know how to talk to the people and they should be exemplary to the people’ (VHT*, *Health facility# 2)*. *‘Healthcare workers are rude so it makes the child to fear if there is too much rudeness from the vaccinators’ (VHT*, *Health facility# 1)*. *‘They (girls) fear these nurses also because these nurses are arrogant…’ (Teacher*, *school in catchment area of Health facility# 3)*.

#### (iii) Community level barriers

*Rumors or misconceptions about the vaccine and vaccination*. All participants reported hearing rumors or misconceptions about the HPV vaccine and vaccination in their communities. Community members were concerned that the vaccine would lead their girls to think they are protected from all sexually transmitted infections and encourage early sexual debut. ‘*Old women used to talk bad again on that vaccine that it is going to spoil girls that when they are vaccinated they will now go and have intercourse thinking that it will protect them from even other sexually transmitted diseases so others they don’t allow girls to be vaccinated’ (HCW*, *Health facility# 8)*.*’ Some of them (community members) say that when you get vaccinated it is going to make you to start having sex at an early age because you will be thinking that you are now so healthy’ (VHT*, *Health facility# 8)*.

Other community members feared potential long-term side effects of the vaccine, which they worried could lead to poor pregnancy outcomes including miscarriages and infertility. ‘*Some are saying these vaccines they give are a root cause of all the reproductive problems like miscarriage*, *some take long to become pregnant’ (HCW*, *Health facility# 5)*. *‘The problem here is the attitude of the local people; others don’t allow their girls to be vaccinated*. *Some it (the vaccine) may deter you from becoming pregnant in future*. *They think it brings infertility’ (Teacher*, *School in catchment area of Health facility# 4)*.

Safety of the vaccine was another reported concern expressed by community members. Rumors circulating in some communities suggested that the vaccine could be a source of other infections like HIV and conditions like diabetes and cancer. ‘*They put HIV in that vaccine so whenever you have been given that one you automatically become sick’ (Teacher*, *school in catchment area of Health facility# 3)*. *‘Some parents say that*, *they (health workers) are vaccinating our children with something that has HIV’ (HCW*, *Health facility# 2)*. *‘Some of them (community members) are complaining about the issue of diabetes maybe it was the effect of the vaccine which is bringing all these ailments*. *Once you are vaccinated and don’t finish the dose the vaccine that is in you could cause another problem to your health’ (Teacher*, *school in catchment area of Health facility# 7)*. *‘And they also say that this vaccine*, *the HPV vaccine that it causes cancer’*. *(HCW*, *Health facility# 7)*.

Community members were also fearful that the vaccine might kill their children out of lack of knowledge about the safety profile of the vaccine. *‘The fear is that some other people do not understand what this vaccine is*. *So they fear that the vaccine might kill their children’ (VHT*, *Health facility# 1)*. Some of the negative perceptions about the HPV vaccine were linked to religious and cultural beliefs. ‘*I have heard some people say that they do not want because the vaccine may be from satanic agents’ (Girl*, *Health facility# 3)*. *‘Other people say that that thing (HPV vaccine) is for people from under the water’ (Caregiver*, *Health facility#3)*. *‘Some parents do not want their girls to be vaccinated*, *some of them say that their children are being vaccinated using the medicine that has been brought from underground’ (Teacher*, *school in catchment area of Health facility# 5)*.

Other community members were reported to express doubt about the efficacy of the vaccine and seemed not to appreciate its benefits. ‘*Others are saying that the vaccine may not work…’ (Teacher*, *school in catchment area of Health facility#8)*. *‘They (community members) say that the vaccine came of recent it was not there those days there is no difference even if you are to get the vaccine or not’ (VHT*, *Health facility# 7)*.

*Strong traditional and religious beliefs*. VHTs and healthcare workers reported that some traditional practices and religious beliefs were against vaccination in general and some of the community members were suspicious that government is using new vaccines for population control. ‘*There are some traditional and religious beliefs that are very negative about vaccination in most cases even if you take the vaccine near them’ (VHT*, *health facility#7)*. *‘They say that for them they do not take their children for immunization because it is against their traditional practices of which when someone is vaccinated they would have broken the rule’ (HCW*, *health facility#5)*.

*Mistrust of government intentions of introducing new vaccines*. Community members were reported to be suspicious about the intentions of government in introducing many new vaccines. *‘People say that those days there were not many vaccines to give to the people…this is a sign that the government is looking for ways of finishing (killing) people by injecting them since they think we are now very many …They (community members) say that Government is overloading their children with the medicine and a lot of things they do not know*. *Some say today you hear over this vaccine …tomorrow again you hear another…so they (caregivers) don’t actually comply’ (HCW*, *health facility#5)*.

*Busy schedules and the gendered nature of care work*. According to reports from healthcare workers some community members preferred to engage their daughters in domestic work or gardening at the expense of letting them go for vaccination. ‘*Some other parents know that their girl is supposed to go back for vaccination today and says me am going to the garden today you remain at home and cook for us food*. *They (caregivers) will just say no*, *no*, *no we are going to the garden to weed*. *The issue of going for vaccination is wasting time*. *I will not allow you’ (HCW*, *Health facility# 1)*. *‘Some parents during some season take their children to the garden especially those who are not in school*. *They see that it is only the garden work that is important’ (HCW*, *Health facility# 6)*.

## Discussion

The objective of this study was to explore barriers to routine HPV vaccination of adolescent girls in a rural Ugandan district. Our study reveals an interplay of barriers to vaccination at individual, health facility and community levels.

We found low awareness of and knowledge about the vaccine among healthcare workers, VHTs, teachers, caregivers and the girls. Sub- optimal knowledge of the vaccine among similar stakeholders has been reported by other studies conducted in Sub-Saharan Africa [22]. What is troubling though, is that the consistently reported low knowledge levels is among healthcare workers and VHTs who have been identified as the most trusted source of vaccine information [23]. Only a few of the interviewed healthcare workers reported attending a condensed one-day training about the vaccine weeks before its introduction in the district and not many VHTs reported formal training about the vaccine. Inadequate training was cited as the primary reason for the sub-optimal level of knowledge about HPV infection, cervical cancer and HPV immunization among healthcare workers in sub-Saharan African countries [24].

The teachers, another potential source of vaccine information for a school-based HPV vaccination program acknowledged inadequate information about the vaccine. The involvement of well-trained school health teams who play a key role in health promotion and social mobilization was found to facilitate the implementation of HPV vaccination programs [25, 26]. With limited knowledge, the girls did not seem to appreciate the benefits of the vaccine and were reported not to take vaccination seriously. Girls who received an explanation of the side effects of the vaccine in a randomized controlled trial were approximately three times more likely to be vaccinated as compared to those who did not receive an explanation [27]. Our findings underscore the need to educate healthcare workers, VHTs and teachers to ensure that they get their facts right about the vaccine and the need to provide information about the vaccine to the girls prior to or during vaccination. Limited awareness and inadequate knowledge fueled community rumors and misconceptions about the vaccine and vaccination. Mistrust of vaccines in general and other health-related products imported from abroad have increased overtime and are common in Africa leading to some resistance of public health interventions. Vaccines are considered a ploy to sterilize or infect non-Western communities [28, 29]. Using interactive communication approaches with parents and communities and early media involvement during the planning phase of vaccination have been found to quickly mitigate rumors and misconceptions [30].

Healthcare workers attributed caregivers’ inadequate information about the vaccine to limited social mobilization and community engagement activities before and during vaccination. Although the newly introduced routine HPV vaccination leverages resources from the existing national immunization program, it is important to note that the HPV vaccination program has unique delivery characteristics when compared to the delivery of routine infant and childhood vaccines. First, the HPV vaccine is relatively new and therefore requires intensive social mobilization and community engagement activities. Secondly, the vaccine is newly introduced into the UNEPI and targets adolescents who are not the usual target for routine vaccination and therefore requires development of strategies and infrastructure to mobilize them. These unique characteristics call for stakeholder collaboration and resources from relevant government ministries including health, education and finance [22] and strengthening UNEPI to effectively deliver the HPV vaccine alongside infant and childhood vaccines. Also required is an intensive communication strategy with culturally appropriate messages to raise awareness about the HPV vaccine, change attitudes of communities about the vaccine and motivate caregivers to encourage their daughters to be vaccinated. Caregivers’ vaccine awareness was a strongest predictor of initiation of vaccine series among ethnic minority adolescents [31]. Parents who were initially reluctant to have their daughters vaccinated because they did not have information about the vaccine became less reluctant as they got more information from trained healthcare professionals [10]. Taken together these findings highlight a need for caregiver education about HPV vaccination. Where intensified social mobilization and community sensitization campaigns have been implemented, there was an increase in demand for vaccination [32].

There were reports that some girls would refuse to be vaccinated without giving any reason and occasionally due to discouragement by peers or caregivers. Where schools are used for vaccine delivery, vaccination may turn into a shared experience influenced positively or negatively by group dynamics especially if the actual vaccination is witnessed by peers [33]. In our study, VHTs reported negative influence by peers, which they attributed to girls’ previous experience with injection pain and lack of the girls’ appreciation of the benefits of vaccination. Poor coordination between healthcare workers and schools was a barrier to vaccination. HPV program evaluations have shown the importance of vaccination programs to be jointly “owned” by both immunization programs and education institutions, for consent, social mobilization, logistics and monitoring [34]. Using a school-based vaccine delivery platform requires careful micro-planning between health and educational officials to facilitate successful delivery [35]. Girls’ absenteeism on vaccination day, high drop out from school or frequent changes of residential location or school within or outside the district between doses were barriers to vaccination as previously reported [36–38]. The addition of an organized reminder to alert the girls or their caregivers about the scheduled date(s) for vaccination could prevent missed opportunities. Additionally, to increase equity for out-of-school girls, additional approaches beyond school-based programs are needed and further research is needed to identify and evaluate potential strategies for reaching out-of-school girls. Overall, high vaccine uptake and high equity is critical for prevention particularly in groups at highest risk of cervical cancer [39].

Impassable roads during the rainy season that lead to poor accessibility to schools disrupted vaccination activities as previously reported for routine childhood vaccination [40] and HPV vaccination in hard- to- reach communities in Kenya [41]. Owing to competing responsibilities at health facilities and several schools to cover, healthcare workers, school authorities and VHTs reported difficulty in conducting active follow-up for girls who missed vaccination as was successfully done by community health workers in Rwanda [42].

Consistent with previous studies, vaccine shortage was a significant barrier, which healthcare workers attributed to insufficient doses delivered by national medical stores to the district [43, 44]. Vaccine stock-outs seemed to be a reflection of bottlenecks in the procurement, transportation and storage of a newly introduced HPV vaccine in the district [45]. Shortfalls in the cold chain infrastructure such as lack of or broken down fridges and shortage of ice packs for the available vaccine carriers were additional barriers consistent with previous studies [38, 46]. However, there seems to be variations in adequacy of cold-chain capacity and infrastructure between districts. Nabirye et al., found a fully functioning cold chain infrastructure in Eastern Uganda, which was attributed to the revamp supported by development partners in preparation for the introduction of other new childhood vaccines [13].

The reported inadequately staffed health facilities are consistent with findings of a recent Ministry of Health audit of human resources for health, which found poorly staffed lower level health facilities [47]. Overstretched and overworked, some healthcare workers were reported to demonstrate disrespectful behavior (unfriendly, talk badly, rude) towards the girls waiting to be vaccinated. As a consequence, disrespectful behavior frightened some of the girls to the point of leaving the health facilities without being vaccinated. Disrespectful behavior of some health professionals has been reported as a major reason for drop out from a childhood immunization program in Ethiopia [48] and a key barrier to HPV vaccination in Eastern Uganda [13]. Our findings highlight the importance of working towards respectful provision of national vaccination services in general in order to improve completion of vaccine doses.

Strong religious and cultural beliefs in opposition to vaccination were reported barriers to HPV vaccination just as they have negatively influenced childhood vaccination programs [49]. There was reported mistrust in government intentions in introducing a new vaccine for girls, which were exacerbated by negative media reports about fake medicines and other sub-standard items on the market. Recent studies from high income countries seem to show that individuals are becoming more skeptical and losing confidence in vaccines, health authorities and government because of the rapid spread of misinformation [50]. Undoubtedly, the provision of scientific information alone will not help reduce uncertainty and anxiety around HPV vaccination [51]. Respecting individuals’ beliefs and lifestyles while providing scientifically sound information by trusted healthcare professionals, including risks and benefits of vaccination is fundamental in helping people understand the rationale and benefits of vaccination for both individuals and communities [52].

Overall, our findings suggest that overcoming barriers to routine HPV vaccination will require strengthening the healthcare delivery system. While national immunization programs are evolving towards a “life-course approach” to immunization by expanding the scope to include adolescents and adults who have previously been underserved, the expansion does not seem to be linked to the necessary health system strengthening initiatives [53, 54]. Active and continuous collaboration between ministries of health and education, community mobilization and sensitization by health workers, the media, teachers, local leaders and community health workers as well as active community follow-up of girls out-of-school and those that miss vaccination at school are key strategies that can help improve vaccination coverage as has been the case in Rwanda [55–57].

### Strengths and limitations

The use of qualitative methods, the triangulation of views of the different categories of participants, and the comprehensive range of questions posed to participants to understand the issues from multiple angles are strengths of our study. However, the findings should be interpreted in light of a number of limitations. First, the use of qualitative methods of data collection did not allow for the quantification of the reported barriers so we cannot estimate how commonly these barriers are faced. Secondly, the findings may not be generalized to other settings especially urban areas since the objective of the study was exploratory and conducted in a single rural district of Uganda. Thirdly, the inclusion of only girls attending public schools, limited subgroup comparison with girls attending private schools. Nevertheless, our results are consistent with findings reported by previous studies done in other low income settings [22, 58, 59] and raise many critical issues yet to be addressed.

## Conclusions and recommendations

Our study suggests an interplay of barriers at individual, health facility and community levels, which prevent routine HPV vaccination of adolescent girls. Strengthening the vaccination program requires providing appropriate information to the girls plus the community, school and health facility stakeholders; addressing cold chain challenges as well as adequate training of vaccinators to enable them respond to rumors about HPV vaccination.

## Supporting information

S1 File Girls Key Informant interview guide–English. (PDF)

S2 File Parents key informant interview guide–English. (PDF)

S3 File HWs and teachers key informant interview guide–English. (PDF)

S4 File Girls key informant interview guide-Luo. (PDF)

S5 File Parents key informant interviews guide–Luo. (PDF)

S6 File HWs and teachers key informant interview guide-Luo. (PDF)
