# Supplementary material for: Why don’t adolescent girls in a rural Uganda district initiate or complete routine 2-dose HPV vaccine series: Perspectives of adolescent girls, their caregivers, healthcare workers, community health workers and teachers
Source: PLoS One. 2021 Jun 29;16(6):e0253735. doi: 10.1371/journal.pone.0253735 (PMC8241119; doi:10.1371/journal.pone.0253735)
Supplement: S1 File — (PDF) [file pone.0253735.s001.pdf]

S1 File. Girls- Key Informant Interview (KII) Guide - English

I. Introduction

Thank you for coming today! My name is .... *INSERT* and I'll be asking you some questions related to health, cervical cancer, HPV, HPV vaccine and your experience with the HPV vaccination. I want you to feel comfortable while we are talking, so don't worry about how you answer the questions, there are no right or wrong answers, we just want to hear your thoughts and opinions. Our talk today will probably last between 40-60 minutes. I would like to record the conversation so we don't miss any of the important things you may tell us. Remember you can refuse to answer any question that makes you feel uncomfortable what is shared during the interview will remain confidential and to the best of our ability, we will not identify anyone specifically. Do you have any questions? …Great, let's get started!

II. Demographics (Record)

1. How old are you?
2. What class are you currently in (e.g. P5)?
3. What school do you currently attend?

III. General preventive perspectives

4. What are some of the reasons you regularly see someone from the health facility (e.g. a health worker or village health team (VHT) member)? Do you think it is better to try to prevent illness, or to treat an illness once you have it? Why do you think so?

5. What are some of the things you do to prevent illness? What does that involve? *Change behaviors?*
6. What are your major sources of information/learning about how to prevent illness?

Probe *for: if not mentioned and type of information obtained*

- *Parents, sibling other relatives*
- *Media – radio , TV, social media, IEC materials*
- *School or teachers*
- *Community members/meetings?*
- *Health facility or health worker?*
- *Peers/friends*
- *Religious leaders*
- *Other? (Probe: church etc)*

7. Think about a time when you did something good for your health that you had never done before or didn't really want to do. What forced you to do it?

IV. Cervical cancer

Now we'd like to talk about cervical cancer.
8. Have you **ever** heard about cervical cancer? *Give a chance to a participant to answer before using the probes below* [**If response is no, tell the participant what cervical cancer is and skip to Q. 14**

9. **If yes:** Tell me what you have heard about cervical cancer?

10. What was your source of information about cervical cancer?
Probe: For all sources of information for the participant and the specific information obtained from:

- *VHT member*
- *Friends or family members*
- *Teachers at school*
- *Health worker*
- *Religious leader*
- *Radio/TV*
- *Other?*

13. Can you tell me about cervical cancer? What is cervical cancer?
12. In your view, what causes cervical cancer? How does a woman get cervical cancer?
13. What can a person do to protect themselves from getting cervical cancer?

V. HPV, HPV vaccine and HPV Vaccination

Now, we'd like to talk about HPV and HPV vaccination

14. Have you **ever** heard about human papilloma virus (HPV)? *Give a chance to a participant to answer before using the probes below* **If no, no tell the participant what HPV is and then go to Q. 24]**

15. What was your source of information about HPV?
P**robe**: from… and type of information obtained

- *VHT member*
- *Health worker*
- *Friends?*
- *family members*
- *Teachers at school*
- *Radio, TV*
- *Other?*

16. Can you tell me about HPV. What is HPV?

17. How is HPV passed on from one person to another? *Give a chance to a participant to answer before using the probes below*
Probe: -
Sharing clothes, -
Mother-to child, -
Sexually transmitted
-Skin-to skin,
- other?

18. What diseases or conditions does HPV cause? *Give a chance to a participant to answer before using the probes below*
Probe:
-cervical cancer?
-Genital warts?
-other cancers? Other infections?

19. In your opinion, what is your chance of getting HPV? What makes you say so?

20. In your opinion, what can you do to protect yourself from getting HPV?

21. Have you ever heard of the HPV vaccine?

22. Can you tell me about HPV vaccine. What is HPV vaccine?

23. How did you hear about the HPV vaccine? *Give a chance to a participant to answer before using the probes below*
Probe*:* from
-VHT member
-Health worker
-Friends Family members
-Teachers at school
-Other?

24. Have you **ever** received the HPV vaccine? (Y/N)
If no, go to Q. 31

25. **If yes**, what month and year did you receive the first dose of HPV vaccine? Where did you get the HPV vaccine? (At school, at the health facility, at outreach post, other?)
26. What disease or condition were you told the HPV vaccine protects you from? Let the girl answer then **Probe** if not mentioned for the following: *Cervical cancer? Genital warts? Sexually transmitted infections? Other?*

27. How many times are recommended by the MoH for a qualified girl like you to get the HPV vaccine? [**If participant does not know, tell her the correct number of times and schedule, then continue with next question.]**

28. How many times did you receive HPV vaccine? **If only once**, why didn't you get the 2nd HPV vaccine dose? **Probe**: Did you transfer schools, did your family move, or did you stop attending school after you received the first vaccine dose, feared injection? Other reason?

29. **If yes to receiving one dose of HPV vaccine:** What was your experience?

30. In your opinion, should girls of similar age like you get the HPV vaccine? (Y/N). Why do you say so?

VI. Perception of HPV vaccine and HPV vaccination

31. What good things have you heard your community members talk about the HPV vaccine? HPV vaccination?

32. What fears or concerns or anxieties have you heard your community members talk about the HPV vaccine? HPV vaccination?

    Probe:
    - Does this vaccine truly prevent HPV infection?
    - Does this vaccine truly prevent cervical cancer?
    - HPV vaccine affects a girl's ability to have children in the future[1]?
    - HPV vaccine increase desire for sex
    - Fear of side effects? Other?

33. Do you think HPV vaccination is important? Why do you say so?

34. Do you know of girls of similar age like you in school who did not get vaccinated? **If yes**, why do you think some of the qualified girls of similar age like you in school didn't get vaccinated?

35. What do you think would encourage or support girls of similar age like you in school to get vaccinated?

36. Do you know some girls of similar age like you in the community who do not go to school? Did they receive the HPV vaccine?
37. **If she knows girls who do not go to school and did not get vaccinated,** what do you think would encourage or support girls of similar age like you who do not go to school in the community to get vaccinated? **Probe:** do you think they would have come to get the vaccine if they were invited to school? Health facility? Home? Outreach post? Other?

38. **If she knows girls who do not go to school and were vaccinated,** how did the girls of similar age like you in the community get information about HPV vaccination?

39. Is HPV vaccination an important topic in your community? What makes you say so?

VII. Health decision-making strategies

We are interested in understanding those who have a role in taking decisions about your health.

40. Who has the most power in making decisions about your health?

*[**Probe**: Yourself, father, mother, sibling, VHT, religious leader, health worker, friend, spouse, other male family member, other female family member, other?]*

41. Does anyone in your family have power to refuse your decision? Who in your family has that refusal power? (**Probe**: Father? Mother? Both? Others?)

42. Do you talk about health issues in your family or make decisions alone?

43. Which family member was involved in your decision making to get the HPV vaccine? How did your parents/guardian help you make the decision to get vaccinated?

44. Who interested you most to get vaccinated? **Probe**: peers, friend, teacher, health worker, VHT member, family member etc?
45. Did your peers or friends or teacher encourage or discourage you to get vaccinated? What encouraging information did your peers or friends or teacher tell you at the time of HPV vaccination? What discouraging information did your peers or friends or teacher tell you at the time of HPV vaccination?[2]?

46. Any other comment about the HPV vaccine? HPV vaccination?
Thank you.
