# Supplementary material for: Why don’t adolescent girls in a rural Uganda district initiate or complete routine 2-dose HPV vaccine series: Perspectives of adolescent girls, their caregivers, healthcare workers, community health workers and teachers
Source: PLoS One. 2021 Jun 29;16(6):e0253735. doi: 10.1371/journal.pone.0253735 (PMC8241119; doi:10.1371/journal.pone.0253735)
Supplement: S2 File — (PDF) [file pone.0253735.s002.pdf]

S2 File. Parents/Guardians -- Key Informant Interview Guide- English

I. Introduction:

Thank you for coming today! My name is .... *INSERT* and I'll be asking you some questions related to healthcare, cervical cancer, HPV,HPV vaccine and your daughter's experience with the HPV vaccination. I want you to feel comfortable while we are talking, so don't worry about how you answer the questions, there are no right or wrong answers, we just want to hear your thoughts and opinions. Our talk today will probably last between 40-60 minutes. I would like to record the conversation so we don't miss any of the important things you may tell us. You do not have to answer any question that makes you feel uncomfortable and the information you tell us will stay confidential and used only for research purposes. Do you have any questions? …Great, let's get started!

II. Demographics of respondent (record)

1. Gender
2. How old are you?
3. What class is your daughter currently in (e.g. P5)?
4. What school does she currently attend?

III. Health and Health care

5. Where do you and your family members normally receive care from when sick?
6. If you or a family member needed medicine or something else important for your health, what would be the easiest way to get what you needed?
7. What are some of the things that make it difficult for you and your family members to seek health care when you need it? When your child needs it?
8. Who makes most of the decisions about your family members' health and healthcare?
9. Do you discuss healthcare decisions as a family? **If yes**, who within the family takes part in these discussions? **If no**, Please explain
10. Who do you talk to when you have a question or concern, worry or seek advice about your health and health care?
11. What health problems are most important to you and your family members? friends?

IV. Cervical Cancer

Now we'd like to talk about cervical cancer.

12. Have you **<u>ever</u>** heard about cervical cancer? [**If said no, tell the participant what cervical cancer is, skip to Q. 14**] **If yes**, tell me what you heard about cervical cancer. **Probe** for information on the following if not mentioned:

- *What is cervical cancer? What causes cervical cancer?* What can one do to protect themselves from getting cervical cancer? **Probe:** *for HPV vaccination; screening (women 25 years and older) other?* if not mentioned.

13. What are your sources of information about cervical cancer? *Give a chance to a participant to answer before using the probes below*

Probe*: from ……and the information obtained from each source*
- *VHT member*
- *friends or family members*
- *teachers at school health worker*
- *Church/community leader*
- *Radio/TV*
- *Read about it in news papers*
- *Other sources*

14. What concern or worries or anxiety have you heard the community members talk about cervical cancer?

15. Is cervical cancer a concern in the community? What makes you say so?

V. HPV, HPV vaccine and HPV vaccination

Now, we'd like to talk about HPV and HPV vaccination

16. Have you **ever** heard about human papilloma virus (HPV)? [**If said no, tell the participant what HPV is and then go to question Q. 22] If yes**, what have you heard about HPV?

17. From what sources of information have you heard about HPV? *Give a chance to a participant to answer before using the probes below*

Probe for*: from ….and information obtained from each source*
-VHT member?
-Health worker?
- Friends?
-Family members?
-Teachers
-Community leaders
-Others?

18. Can you tell me about HPV? What is HPV?
19. How is HPV passes on from one person to another? *Give a chance to a participant to answer before using the probes below*
Probe:
-Sharing clothes,
-Mother-to child,
-Sexually transmitted
-Skin-to skin,
- Blood transfusion
-Other?

20.What diseases or conditions does HPV cause? *Give a chance to a participant to answer before using the probes below*
Probe:
-cervical cancer?
- Genital warts?
- Other cancers? Other infections?

21. In your opinion, what is the chance of your daughter getting HPV? What makes you say so?

22. In your opinion, what can you do to protect her from getting HPV?

23. Have you ever heard of the HPV vaccination? [**If said no, tell the participant what HPV vaccination is, skip to Q. 26**] **If yes**, what have you heard about HPV vaccination?

24. What are your sources of information about HPV vaccination? *Give a chance to a participant to answer before using the probes below*

Probe: from **……**and information obtained from each source
- *VHT member*
- *health worker*
- *friends*
- *family members*
- *teachers at school*
- *Radio/TV*
- *Community/religious leaders*
- *Other?*

25. Which groups of people/individuals does the MoH recommend to receive the HPV vaccine? **Probe**: In-school which group? What age? and Out-of school..which group? what age? **[If participant does not know, tell them the recommended qualified in-school and out-of school individuals and age(s)]**
26. How many doses of HPV vaccine and at what interval does the MoH recommend a qualified person get vaccinated in order to be fully protected against HPV? **[If participant does not know, tell them the number of recommended number of doses and intervals for the HPV vaccine]**
27. What venues are normally used for HPV vaccination exercise? **Probe** if not mentioned..schools, health facility, outreach posts, Others?
28. Do you know any individuals or groups of people in your community that have been vaccinated against HPV? **If yes** which individuals or groups? When did the most recent HPV vaccination take place in your community? (**Probe for:** month, year) if not mentioned
29. Do you think HPV vaccination is important to the community?
30. Did your daughter receive the HPV vaccine? **If no**, why didn't your daughter receive the HPV vaccine? **Probe if not mentioned:** refused vaccination, vaccines got finishes (stock out), was absent from school…

If yes:
31. How many doses of HPV vaccine did your daughter receive?
32. Where did she receive the vaccination from?

***Probe**: school, health facility, outreach post, other?.... if not mentioned*
33. Where would you have liked your daughter to get vaccinated?
Probe: *school, health facility, outreach post, at home, other?* Why would you prefer the above place?
34. What was your experience with your daughter's HPV vaccination?
35. Are you happy that your daughter got vaccinated? Please explain why? What did she tell you about her experience with the HPV vaccination? **Probe**: for experience before and after vaccination
36. Do you have any worries, concerns or anxiety about your daughter getting vaccinated? If yes, what are they? *(Please explain in the order of priority)*
37. Do you know girls of your daughters age in school who were not vaccinated? **If yes**, what ideas/suggestions do you have to ensure that qualified girls in school receive the 2-doses of the HPV vaccine?
38. Do you know girls of your daughter's age who do not go to school in the community? What ideas/suggestions do you have to ensure that qualified girls in the community who do not go to school receive the 2-doses of the HPV vaccine? **Probe**: Do you think they would come to get the HPV vaccine if they were invited to school? Health facility? Outreach post? Other? Why do you think so?
39. In your opinion, what would be the right place for girls of similar age to your daughter who do not go to school in the community to get vaccinated ? **Probe**: *School, Health facility, outreach post, other?*
40. In your opinion, what would be the best way to reach girls of similar age like your daughter with information about the HPV vaccine? HPV vaccination?(**Probe** if not mentioned: phone call to the girl? Parent/guardian? Health worker/VHT member, text message to the girl? Parent/guardian ?Health worker/VHT member? Other?)

41. Any other comment about HPV vaccine? HPV vaccination?

VI. Perception of HPV vaccination

42. What good things have you heard your community members talk about the HPV vaccine? HPV vaccination?
43. What fears or concerns have you heard your community members talk about the HPV vaccine? HPV vaccination?
Probe: *if not mentioned*
-Vaccine may not be effective to prevent HPV infection
-Vaccine may not be effective to prevent cervical cancer
-The vaccine may prevent girls from giving birth to children
-Vaccine will make girls sexually active
-Other concerns?

V. Decision making

44. Can you tell me what your daughter told you about HPV vaccine?
45. Who helped your daughter to make a decision to get vaccinated?
Probe: *health workers, VHT, teacher, friends, an elder, other?*

Thank you.
