# Supplementary material for: Why don’t adolescent girls in a rural Uganda district initiate or complete routine 2-dose HPV vaccine series: Perspectives of adolescent girls, their caregivers, healthcare workers, community health workers and teachers
Source: PLoS One. 2021 Jun 29;16(6):e0253735. doi: 10.1371/journal.pone.0253735 (PMC8241119; doi:10.1371/journal.pone.0253735)
Supplement: S3 File — (PDF) [file pone.0253735.s003.pdf]

S3 File. Teachers/Healthcare Providers/Vaccinators -- Key Informant Interview Guide- English

I. Introduction:

Thank you for coming today! My name is .... *INSERT* and I'll be asking you some questions related to health, cervical cancer, HPV, HPV vaccine and HPV vaccination. I want you to feel comfortable while we are talking, so don't worry about how you answer the questions, there are no right or wrong answers, we would like hear your thoughts and opinions. Our talk today will probably last between 40-60 minutes. I would like to record the conversation so that we do not miss any of the important things you may tell us. You do not have to answer any question that makes you feel uncomfortable and the information you tell us will be kept confidential and used only for study purposes. Do you have any questions? …Great, let's get started!

II. Demographics (Record)

1. What is the name of health facility you are attached to/work?
2. What is your current position?
3. How long have you served in your current position?
4. What is your highest level of education?
5. Gender
6. How old are you?

III. Health and General sources of health information

7. Tell me about the major health concerns in your community today? **Probe:** for concerns of men and women; boys and girls
8. What health topics do you think are most important to your community?
9. What are the major sources of health information in your community? **Probe**: for men? Women? Boys? Girls?

IV. Knowledge and communication about Cervical Cancer:

10. Have you ever heard about cervical cancer?
11. **If yes:** Tell me what you have heard about cervical cancer
    If no, *tell participant what cervical cancer is and skip to* ***Q. 13***
12. What was your source of information about cervical cancer?
    Probe*: from……and the type of information obtained*
    - *VHT member*
    - *friends or family members*
    - *teachers at school health worker*
    - *Church/community leader*
    - *Radio/TV*
    - *Read about it in news papers*
    - *Other sources*
13. In your view, what causes cervical cancer?
14. Is cervical cancer a concern in your community?
- **If yes** why is cervical cancer a concern?
- **If no** why is cervical cancer not an issue of concern in your community?
15. What information have you heard your community members talk about cervical cancer?
16. What concerns or worries have you heard your community members talk about cervical cancer?

17. In your view what can be done to prevent cervical cancer? **Probe** for: HPV vaccination (girls); screening (women 25 years and older), other? if not mentioned

V. HPV and HPV vaccination:

18. Have you <u>**ever**</u> heard about human papilloma virus (HPV)? *(If no, give description for HPV and go to* **Q. 22)**
19. **If yes**, can you tell me what you have heard about HPV? What is HPV?
20. What was your source of information about HPV? **Probe***: from……and the type of information obtained from each source*
    - *VHT member*
    - *friends or family members*
    - *teachers at school health worker*
    - *Church/community leader*
    - *Radio/TV*
    - *Read about it in news papers*
    - *Other sources*
21. How does a person get HPV? **Probe** if not mentioned…
    -Sexually transmitted
    -Skin- to- skin
    -Sharing clothes
    -mother-to child
    -Blood transfusion
22. What diseases or conditions does HPV cause?
    Probe:…. if not mentioned
    -cervical cancer?
    -Genital warts?
    -other cancers? Other infections?
23. Have you <u>**ever**</u> heard about the HPV vaccine?
24. **If yes**, can you tell me what you have heard about HPV vaccine? What is the HPV vaccine? **If no, tell the participant what the HPV vaccine is, skip to Q. 26**
25. What was your source of information about the HPV vaccine? **Probe***: from..* and the nature of information provided about HPV vaccine *from each source*
    - *VHT member*
    - *friends or family members*
    - *teachers at school health worker*
    - *Church/community leader*
    - *Radio/TV*
    - *Read about it in news papers*
    - *Others*
26. Do you think the HPV vaccine is important to your community? What makes you say so?
27. Have you participated in HPV vaccination exercise in the past 12 months? **If yes**, which months?
28. What is the MoH model of delivery of HPV vaccine in the district?
    Probe if not mentioned..(i)Routine i.e. throughout the year (ii) during child days plus months (iii) Routine but particularly during child days plus months **[If participant does not know, tell them the MoH's model of delivery for HPV vaccine]**

29. Which groups of people/individuals does the MoH recommend to receive the HPV vaccine? **Probe**: In-school which group? What age? and Out-of school..which group? what age? **[If participant does not know, tell them the in-school and out of school individuals and age(s)]**
30. How many doses of HPV vaccine and at what interval does the MoH recommend a qualified individual to get vaccinated in order to be fully protected against HPV? **[If participant does not know, tell them the number of doses and interval recommended by the MoH]**
31. What venues are normally used for HPV vaccination exercise? **Probe** if not mentioned..schools, health facility, outreach posts, Others?
32. Do you know any individuals or groups of people in your community that have been vaccinated against HPV? **If yes** which individuals or groups? When did the most recent HPV vaccination take place in your community? (**Probe for:** month, year) if not mentioned
33. Why do you think some girls suitable for HPV vaccination in your community don't get the (i) 1st dose and (ii) 2nd dose of HPV vaccine? **Probe for……. If not mentioned**
    individual (lack of information, low perceived risk of getting HPV-not yet sexually active, fear of injection, fear of side effects)
    Family (lack of information, general parental attitudes and belief about vaccines, concerns about safety and long term effects of the vaccine e.g. infertility, promiscuity)
    Community (suspicious of vaccines made in Western countries)
    Health system factors (lack of community mobilization and sensitization for vaccination exercise, long distance to vaccination site, inadequate vaccine).
34. What do you think would make it easier for qualified girls who do not go to school in your community to get the 1st dose (ii) return for the 2nd dose of HPV vaccine?
35. Why do you think some qualified girls in school do not get the 1st dose and (ii) return for the 2nd dose of the HPV vaccine? **Probe for……. If not mentioned**
    individual (lack of information, low perceived risk of getting HPV-not yet sexually active, fear of injection, fear of side effects)
    Family (lack of information, general parental attitudes and belief about vaccines, concerns about safety and long term effects of the vaccine e.g. infertility, promiscuity)
    Community (suspicious of vaccines made in Western countries)
    Health system factors (lack of community mobilization and sensitization for vaccination exercise, long distance to vaccination site, inadequate vaccine).
36. What would make it easier for qualified <u>**girls in school**</u> to get the first dose of HPV vaccine? Return for 2nd dose of HPV vaccine?
37. What would make it easier for qualified <u>**girls out of school**</u> in the community get the 1st dose of HPV vaccine? Return for the 2nd dose of HPV vaccine?
38. What good things have you heard the community talk about the HPV vaccine? HPV vaccination?
39. What concerns or worries or anxieties have you heard the community talk about the HPV vaccine? HPV vaccination?
    Probe: fear of infertility, increasing sexual desire, side effects, others? if not mentioned
40. Have you heard about any medical problems or side effects in the community following the most recent HPV vaccination exercise? **If yes**, describe the medical problems and what happened?
41. In your opinion, is (i) cervical cancer (ii) HPV vaccine and (ii) HPV vaccination important topics in your community? What makes you say so?

Promotion of HPV Vaccination

42. What do you think are the benefits of HPV vaccination?
43. What do you think would be the most helpful way to educate girls and their parents /guardians about the benefits of the HPV vaccination?
44. What do you think would be the most helpful way to promote HPV vaccination to suitable girls in the community? Their parents/guardians? The health workers/VHT vaccinators? Teachers? **Probe for the following if not mentioned**:

-Posters/ fliers in health facility or community center, or school or community church
-A talk from a respected person at a community church or local radio station or other community-based organization
-Send timely text message to the parent/guardian? Girl? as a reminder via mobile phone
-Educate parents/guardian and girls about HPV vaccination by sending text messages through mobile phone
-Send email information or text message reminders to health workers and VHT vaccinators?
--Health fair with information about how and where to get vaccinated
-A talk from health worker to eligible girls and their parents/guardians at school
Other community setting?

44. What do you think are things that could be done differently (i) <u>before</u> and (ii) <u>during</u> the HPV vaccination exercise that would encourage all suitable **<u>girls in school</u>** to get vaccinated? **Probe for the following if not mentioned**:

- Health worker and teacher education, training and communication about HPV vaccine
- Education of suitable girls before and/or during HPV vaccination
- Improvement in coordination and communication between school and qualified girls and vaccinators
- Improvement in the distribution of HPV vaccine supplies and logistics,
- Improvement in staff transportation to venue of vaccination,
- Increase in parental/guardian participation in HPV vaccination exercise
- Increase in teacher participation in HPV vaccination exercise
- Timely payment of staff allowances
- Other?

45. What do you think are the things that could be done differently before and/or during the HPV vaccination exercise that would encourage all suitable **<u>girls out of school</u>** in the community to get vaccinated?

Probe for the following if not mentioned:

- Health worker training and communication about HPV vaccine and HPV vaccination
- Education of suitable girls before and/or during HPV vaccination
- Improvement in coordination and mobilization of out-of school girls and their parents/guardians by VHTs
- Improvement in the distribution of HPV vaccine supplies and logistics,
- Improvement in staff transportation to venue of vaccination,
- Increased parental/guardian participation in HPV vaccination exercise
- Timely payment of staff allowances
- Other?

46. What do you think are some of the main reasons that get in the way of a girl who gets the first dose of HPV vaccine from getting the second dose? **Probe for the following if not mentioned:**

- Parent/guardian do not give permission to be vaccinated

- Girl refuses to be vaccinated
- No documentation in HPV register by staff involved in vaccination exercise
- Absent from school on the day of vaccination
- Changing schools
- Drop out of school
- Vaccine out of stock
- Fear of injection
- Fear of side effects
- Other?

47. What do you think should be done to ensure that all qualified girls who get the first dose of HPV vaccine return to receive the second dose of HPV vaccine?
48. Any other comment about HPV vaccine? HPV vaccination?

Thank you.
