# Supplementary material for: Why don’t adolescent girls in a rural Uganda district initiate or complete routine 2-dose HPV vaccine series: Perspectives of adolescent girls, their caregivers, healthcare workers, community health workers and teachers
Source: PLoS One. 2021 Jun 29;16(6):e0253735. doi: 10.1371/journal.pone.0253735 (PMC8241119; doi:10.1371/journal.pone.0253735)
Supplement: S4 File — (PDF) [file pone.0253735.s004.pdf]

S4 File. Girls- Key Informant Interview (KII) Guide Luo

I. Tuce/wiye wiye

Apwoyo in me bino tin! An nyinga .... *KET NYINGI KANY* abipenyo in lapenyo in I lokkumyotokum, two akwota ma makodokotnywal pa mon, yat HPV kingecma in I tyekwede ma lube in gwerekiyat HPV ma gengo two akwota ma makodokotnywal pa mon. An amitoniiwinybagonyakacewatyekanywakolokmon, pe I bed kipaaiyoomokeken kit ma omyero I gam kilapenymokeken. Petyelagammo ma tyekakareonyorac, watyekimiti me winyotamionyo kit ma in I neneokwedelok man. Lokwamatintweroterodakika ma room 40-60. An abimakodwonwa I kare me nywako tam man wek pew ii waowil I komlokmo ken ma piretektutwal ma in ibi Waco it wa. I tyekitwerope me gamo kit lapenymokeken ma peiwinyoagonya me gamo ne dokbeneki t lokmokeken ma in ibi Waco I wan bi gak I mwung ma ngatmoatarpe bi ngeyo ne. Ityekilapenymokeken?.....Ayellamopeke, wayaplokwa ma tin!

II. Lok kum in

1. Mwakaniadii?2.
2. Itye I kilaciadii (e.g. P5)?
3. Itye ka kwan I gang kwan kwene?

III. Kit ma dano neno kwede kit me gengo ne.

4. Pi kit lok ango ma oweko I neno latic me yot komk iot yat dik dik ni? (labo ne daktar onyo latic me yot kom ma tye I kin gang kany? Inongoni opore me yelle me juko onyo gengo two, onyo me cango ne kace itye kwede? Pingo I tamo kit meno?
5. Kit gin ango ma in itimo me gengo two pe me maki? Man kwako timo gin ango? Lok kit in calo dano adana?
6. inongo ngec tutwalle kikwene/pwonye iyoo ma pat pat me gengo two?

   Peny ma tut I kom:
   - *Lunyodo ne, omega ne kilumegi ne wakiwadi ne*
   - *Ngec ma nonge I yamo – radio , TV, internet calofacebok, IEC materials*
   - *Gangipwonyoonyolupwoye*
   - *Dano ma I kabedo ma orumowa/ kacoke ma pat pat?*
   - *Otyatonyodaktari?*
   - *Lu rem?*
   - *Lupwonyedini?*
   - *Peny ma tut? (openykacetye: gang legaonyoyoomo ma pat)*

7. Tam kare mo ma in itimo gin mo maber me kony pi yot kom in ma pud manaka yang pud peya pi in onyo I mito timo ne. Ngo ma odiyo in me timo ne?

Miti me nongo agwera ki yat lageng kwidi HPV ki ngo ma tye ka gengo bulu ma anyira ma gitye I caro I Uganda me nongo agwera man

IV. kanca ma makodokotnywal pa mon(Cervical cancer)

I cawa ma amito lok ikum two akwota/kanca ma mako dok otnywal pa mon onyo cervical cancer.

8. In dong tika I winyo lok mo keken I kum two akwota/kanca me dok ot nywal pa mon?*kong I mii kare ki ngat ma me gamo lapeny ma pud pe iyabo penyo ne ma tut ki lapeny ma piny magi*[**kaceowaco nip e iwinyo kit lok mo keken, tit tite ngo ma two kanca me dok ot nywal pa mono bedo ci I mede ki ..tika I winyo/ineno lok acoya mo me gengo two kanca me dok ot nywal pa mon*(cervical cancer)….*]**
9. **kace owaco ne I winyo/ I neneo:**waca ngo ma in iwinyo ikum two kanca me dok ot nywal pa mon?
10. I nongo ngec man ikom kanca me dok ot nywal pa mon kikwene?

    Peny ma tut: onongo ngec man I kom kanca ma mako dok otnywal pa mon ni kikwene ki kit nyik ngec acel acel ma onongo ki bot:

    - *Laticyotokum ma ikingangi (VHT member)*
    - *Luremonyowadi ne*
    - *Lupwonye ma I gang kwan*
    - *Laticyotkum me otyat*
    - *Lutelladini*
    - *Radio/TV*
    - *Yoo ma pat?*

11. I twero tita lok mo manok ikum kanca me dog ot nywal? Kanca ma me dog ot nywal pa mon obedo gin ango?
12. Kit ma in I neon kwede, ngo ma kelo kanca me dok ot nywal pa mon?Dako nongo two kanca man me dok ot nywalni nining?
13. Gin ango ma ngat acel acel twero timo me neon ni gi gwoke ki ikum nongo kanca me dok ot nywal pa mon?

V. Kwidi HPV ki gwere ki yat me gengo kwidi HPV

I cawa man, amitolok I kum HPV kigwereki yet me HPV

14. Tika dong inongongecmoikumkwidi ma kelo two kanca me dokotnywal pa mon (HPV) ?*mi it ngat ma kare me gamolapeny man ma pud me penyo en lapeny me nianglok ma tut magi* ***[**Kaceowaconipeinongo, wac it ngat man ngo ma kwidi HPV obedo ci imedekilapeny Q17]**
15. **I nongongec man kikwene I kumkwidi HPV?**
    - **Peny ma me niang ma tut**: ki bot
    - *La memba me VHT*
    - *Laticyotkom*
    - *Larem?*
    - *wat*
    - *lupwonye I gang kwan*

Miti me nongo agwera ki yat lageng kwidi HPV ki ngo ma tye ka gengo bulu ma anyira ma gitye I caro I Uganda me nongo agwera man

- *Radio, TV*
- *Onyoiyoomukene ma pat?*

16. Itwerowacango ma in ingeyo I kum HPV ? HPV obedo gin ango?

17. HPV kobo mi aa ki ikumn gat acel me ceto I kum ngat mokene nining? *Mi kare ki la ngat man me gamo lapeny me pud me I penyo lapeny me niang ngec ma tut ki lapeny ma piny magi.*

    Peny mi niang ma tut:
    -Leyobongi,
    -Min latin bot latin,
    -Kobo Iyoo me rwate I butu
    -ka del kumaojwaanekikomngatmukene,
    - iyoo ma pat ki magi?

18. Kwai two onyo peko ango ma kwidi HPV kelo? Mii kare kin gat man me gamo lapeny man ma put pe iyabu penyo ne mi niang ngec ma tut kila peny magi:**Peny me niang ngec ma tut:**kanca

    - me dog ot nywal pa mon?
    - Two akwota ma mako kum wa me coo onyo me mon?
    - kwaikanca ma pat?

19. Ki itamni, I nenocaloitweronongokwidiHPV? Ngo ma weko I amo kit man?

20. Kit ma in ineno kwede, Ngo ma in itwero timo ne me gwokeki I kom kwidi HPV?

21. Tike dong I winyo pi gwere I kit yat ma gengo in nongo kwidi HPV?

22. Lok ango ma itwero Waco it an ma kwako yat HPV ? yat HPV obedo gin ango?

23. I winyo pi gwere ki yat HPV nining? *Mii kare ki ngat man me gamo lapeny man mot ma pud pe I penyo lapeny ma piny magi mi ninang lok ma pol.*

    Peny I kum jami magi*:* I kum
    - lumenba me *VHT*
    -Lutic yoto kum I ot yat
    -Lurem
    -Wadi ?
    -Lupwonye I gang kwan?
    -Onyo yo ma pat?

24. In dong inongo gwer me yat HPV?(Y/N)

Kace owaco ni pud, mede ki lapeny nama. 31

25. Kace owaco ni inongo, I dwe ki mwaka mene ma inongo dose me aceI me agwera me yat lageng kwidi HPV? I nongo yat man ki kwene? (gang wan, ot yat, I yub me me kin gang, kamu kene?)
26. Two ki goro kum ango ma ki Waco it in ni yat agwera me lageng kwidi HPV obi konyo in pe me nongo ne? wek nyako man ogam lapeny **Peny me niang ma tut** kace pe owaco mo keken ki ikin dull magi: kanca me dok ot nywal pa mon? aloba loba me kum me mon? two ma kobo iyoo me rwatte I bwuttu? Kwai two ma pat ki magi?

Miti me nongo agwera ki yat lageng kwidi HPV ki ngo ma tye ka gengo bulu ma anyira ma gitye I caro I Uganda me nongo agwera man

27. Minicta me yot kum (MoH) Waco ni anyira calo in opore mi ningo gwer ki yat HPV tyen adii?[**kace ngat man pe ngyeyo, Waco tyen adii ki kume ki Waco ite kare mene man en twero nongo gwer magi ci mede ki lapeny mukene.**

28. Inongo gwer ki yat HPV dong tyen adii? **Kace tyen acel keken**, pingo pe inongo dose gwer me aryio ma odong ni?

Peny mi niang ma tut: I kobo gang kwan, odak adaka ,keje I juku kwan woko ingee nongo dose me acel me yat HPV, ilworo ni kituc in ki lubira? pi tien lok ma pat?

29. **Kace I nongo dose me acel me gwer me yat HPV.** Gwer man owoto ni nining?

30. Kit ma in itamo kwede, itamo ni anyira ma mwaka ki rom onyo cok cok ki mwaka ni omyero gu nong gwer me yat lageng kwidi HPV?(Y/N). Pingo I waco kit meno?

VI. Kit ma ki neno kwede gwer ki yat lageng kwidi HPV

31. Ngo ma in iwinyo ma ber ma lwak ma gi tye I kabedo man gi loko maber ma lube ki yat me gwere HPV? Gwere ki yat HPV?

32. Kit lok ango a in iwinyo lwak ma tye I kabedo man loko marac onyo ma poto cwiny dano ma lube ki gwer me Yat HPV? Gwer kit yat HPV?

    Peny me niang ma tut:
    - Yat man mono gengo kwidi HPV me mako anyira matino?
    - Yat ma mono ada gengo two kanca me dog ot nywal pa mon?
    - Kwidi HPV mono dwoko piny kero pa anyira me nongo lutino in anyim?
    - Yat HPV momo medo miti pa anyora me rwate in buttu
    - Tyen lok ma pat?

33. I neno calo gwer ki yat HPV mono pire tek? Pingo I tamo it meno?

34. I ngeyo anyira ma mwaka ki rom ki megi onyo cok cok ki mwaka ni ma pugu nongo gwer me yat HPV in gang kwan ma in ikwano iyee?

35. Ngo ma in ineno ni kace kitimo obi medo miti onyo modo miti me gwere ki yat HPV in kin anyira I gang kwan man ma in I kwano iyee?

36. I ngeyo anyira ma mwaka gi cok onyo rom ki megi ma gi tye ganag ma pe ki cito I igang kwan? Gin dong gu nongo gwer me yat HPV?

37. **Kace nyako man ngeyo anyira ma pe gunongo agwera dak bene pe gi ceto I gang kwan,** ngo ma in itamo ni omyero ki tim wek anyira magi ma mwaka gi rom onyo cok cok ki megi ma tye gi bedo gang lapongo ceto I gang kwan wek gunong agwera ki yat lageng kwidi HPV ? ***Peny me ninag ma tut*****:** itamo ni onongo gin gi obi bino ka nongo ne I gang kwan kace no ki lwongo gi I gang kwan? Odi yati? Gangi? Ki akwano ma ki keto iteng gudi? Yoo ma pat?

38. **kace ngeyo anyira ma pe kwano ento gu nongo agwera,** angira ma mwaka gi cok cok ki mwaka ni ginongo ngec nining I kum Gwer ki yat lageng kwidi HPV?

Miti me nongo agwera ki yat lageng kwidi HPV ki ngo ma tye ka gengo bulu ma anyira ma gitye I caro I Uganda me nongo agwera man

39. nongo agwera ki yat HPV mo tye gin ma pire tek I kabedo ma I yaa ki iyee?

VII. Moko tam ma kwako yot kum:

Watye ki miti me ninag joo ma gitye ki tic me moko tam ikuk jami onyo lok ma kwako yoto kum in.

40. Anga ma tye ki twero matek me moko tam ikum jami ma kwako yot kum in ma lube ki yoto kum in?

*[**Peny me niang ma tut**: in, baba ni, mamani, lamero onyo omero, VHT, lutela dini, latic yot kum, larem, slu ota, coo ma tye gang, wat mokeken, ngat mo kener?]*

41. Ngat mo tye gang ma tye ki twero me kwero tam ma in I moko piri keni? Anga man ma tye ki twero man?(peny mi niang ma tut: baba?mama? gin weng? Ngat mu kene?)

42. I nywako tam ma kwako yot kum in ki dano gang wu? Keje I moko tam man piri keni?

43. Anga ki gang wu okonyo I ki moko tam me nongo gwer ki yat HPV? La nyodo ni okonyo in nining me moko tam man me nongo gwer me yat HPV?

44. Anga ma omiyo it in mitie nongo gwer me yat HPV? **Peny mi niang ma tut:** jo ma imaro bedo kwed gi, luremi, lupwonye I gang kwan, lutic yoto kum, wadi ni, onyo ngat ma pat?
45. Luremi mono bedo ka miyo it in miti onyo kwanyo cwiyi ki iku nongo kwer man me HPV? Lok mene me miyo miti ma gin gu Waco it in I kare me nongo gwer man? Lok mee ma luremi gu Waco it in ma obedo ka kwanyo cwinyi I kum   nongo gwer me yat HPV?

46. I tye ki kit lok mo keken ma kwako nongo gwer ki yat HPV?

Apwoyo matek.
