# Supplementary material for: Why don’t adolescent girls in a rural Uganda district initiate or complete routine 2-dose HPV vaccine series: Perspectives of adolescent girls, their caregivers, healthcare workers, community health workers and teachers
Source: PLoS One. 2021 Jun 29;16(6):e0253735. doi: 10.1371/journal.pone.0253735 (PMC8241119; doi:10.1371/journal.pone.0253735)
Supplement: S5 File — (PDF) [file pone.0253735.s005.pdf]

Miti me nongo agwera ki yat lageng kwidi HPV ki ngo ma tye ka gengo bulu ma anyira ma gitye I caro I Uganda me nongo agwera man

S5 File. Parents/Guardians -- Key Informant Interview Guide- Luo

I. Tuce/wiye wiye:

Apwoyo in me bino tin! An nyinga ....*KET NYINGI KANY* abi penyo in lapeny in I lok kum yoto kum, two kanca/akwota ma mako dok ot nywal pa mon, yat me gengo kwidi HPV ki ngec ma in I tye kwede ma lube gwere ki yat HPV ma gengo two akwota ma mako dok ot nywal pa mon. Alego in me winyo agonya kace watye kanywako lok man, pe I bed kipaa iyoo mo keken kit ma omyero I gam kila peny mo keken. Petye lagam mo ma tye kakare onyo rac, watye ki miti me winyo tami onyo kit ma in I neon kwede lok man. Lok wa man tin twero tero dakika ma room 40-60. An abi mako dwon wa I kare me nywako tam man wek pe wii wa owil I kom lok mo ken ma pire tek tutwal ma in ibi Waco it wa. I tye ki twero pe me gamo kit lapeny mo keken ma pe iwinyo agonya me gamo ne dok bene ki t lok mo keken ma in ibi Waco It wa bi gak I mwung ma ngat mo ata pe bi ngeyo ne. Itye ki lapeny mo keken?.....Ayellamopeke, wayaplokwa ma tin!

II. Lok angeya ma kwako kom lanyodo (mak dwone)

1. Dako onyo laco
2. Mwaka ni adii?
3. nyari kwana kilaci adii kum kare ni? (e.g. P5)?
4. Anyaki ni man tye ka kwan I ganga kwan kwene?

III. Nongo kony me yot kum

5. Kwene man in ki joo gani ongongo iyee kony me ot yat kace tye two tye?
6. kace in onyo dano mo ma gangi miti yat onyo kony mo me yot kum, yoo mene ma in I neno ni obedo yoo ma yot me nongo kony man?
7. Kwai jami ango ma I neno waci tye ka weko bedo tek tutwal ki joo ma gangi me nongo kony me yot kum kace gi mito nongo ne? Kace latini mito kony me yot kum?
8. Anga ma moko tam ma kwako yoto dano ma in gang wu?
9. Onywako tam ma kwako yot kum gang wu ki dano duc ma gang karacel?kace onywako tam ka acel, anga gi ma bedo ka nywako tam man? Kace pu onywako tam, pingo pu onywako tam ka cel?
10. anga ma I loko kwede kace I tye ki koko onyo adiya adiya mo, cwer cwiny onyo imito nongo kony ma kawako yoto kum in?
11. Kit peko me yoto kum mene ma pire tek tutwal I dye kal wu onyo bot in? Luremi?

IV. Kanca me dog ot nywal pa mon (Cervical Cancer):

I cawa man amito ni wa lok kong I kum kanca me dog ot nywal pa mon onyo cervical cancer.

12. Tika dong I winyo kit lok/ngec mo keken ikum kanca me dog ot nywal pa mon? **[kace petye ki ngec mo keken, wac ite ngo ma kwidi HPV obedo ci mede ki lapeny Q14**] kace I winyo, Kong I

wac it an lok ango ki kume ma iwinyo ikum kanca man me dog ot nywal pa mon ni. **Peny mu niang ma tut ikum kwai nyik lok magi kace pe owaco gi:**

- *Kanca me dog ot nywal pa mon(cervical cancer) obedo gin ango?*
- *Ngo ma ngat acel acel twero timo ne me gwoke ki ikum nongo kanca me dok ot nywal pa mon?**peny mi niang ma tut** ikum nongo agwera, pime (mon ma mwaka oyabe 25 odok malo) yoo ma pat kace pe ki Waco.*

13. I nongo ngec I kum kanca me dog ot nywal pa mon ki kwene? *Mi kare kin gat ma me miyo lagam ma mege me pud peya I mede ki penye me niang lok ma tut ki lapney magi ma piny ni [**kace owaco ni pe ingeyo, tit ite ngo man kanca me dog ot pa mon obedo ci mede ki lapeny mukene]***

    Peny ma tut*: ki bot*
    - *Lamemba pa VHT*
    - *lurem onyo wadi ma gang*
    - *lupwonye I gang kwan onyo lutic yot kum*
    - *lutella dini/ lutella tedero*
    - *Radio/TV*
    - *Okwano I gazette*
    - *Onongo ki kamo kene*

14. lok ango ma rac ma I winyo dano loko ma rac ikum kanca me dog ot nywal pa mon I kabedo ma in iaa ki iyee?
15. Kanca me dok ot nywal pa mon mono ki neon calo gin ma pire tek I kabedo me I aa iyee? Pingo I Waco kit menio?

V. kwidi HPV ki Gwere ki yat lapgeng kwidi HPV

I cawa man, wabi loko I kum kwidi HPV ki kwere ki yat lageny kwidi HPV

16. I tika dong I winyo lok mo ikum kwidi HPV? **[kace owaco nip e iwinyo, tuc it ngat ma ngo ma kwidi HPV obedo ci mede ki lapeny Q.22]** kace owinyo, lok ango ma iwinyo*?*
17. Iwinyo lok onyo ngec ikum HPV ki kwene? *Mi it ngat man kare me gamo lapeny man ma pud peya penye mi niang lok ma tut ki lapeny ma piny magi* [**kace owaco nip e iwinyo kit lok mo keken, Waco it en ngo ma kwidi HPV obedo ci I mede ki lapeny nama Q.22 ma loko ikum Gwer ki yat lageng kwidi HPV**]

    Peny mi niang ma tut*: ki bot*
    - *Lamemba pa VHT*
    - *lurem onyo wadi ma gang*
    - *lupwonye I gang kwan onyo lutic yot kum*
    - *lutella dini/ lutella tedero*
    - *Radio/TV*
    - *Onongo ki iyoo mukene*
18. Kong iwac it an ngo ma in ingeyo ikum kwidi HPV? Kwidi HPV obedo gin ango?

19. Kwidi HPV mono kobe nining ikum dano? *Mi kare ki ngat man mi gamo lanpeny ma pud pe ipenyo mi niang ma tut.*

    Peny mi niang ma tut:

    -leyo bongo

    -Min latin mi ceto ikum latin,
    -rwatte ibutu

    -kudu kum,

    -kobo iyoo ma pat?

20. Kit two onyo goro kum ango ma kwidi man kelo ? *mi kare ki ngat man mi gamo lapeny ma pud peya iyabu penyo en mi niang ma tut ki lapeny ma piny magi*:

    Peny mi niang ma tut ikum:

    - Two kanca me dog ot nywal pa mon?
    - aloba loba ikum me mon?
    - kwai kanca mukene ma pat?

21. Ki itam ma megi, I neno calo nyari twero nongo kwidi HPV? Ngo ma weko in I Waco kit meno?

22. Ki itam ma megi, ngo ma in ineno calo itwero timo nen wek latinin man pu onong kwidi HPV?

23. Tika dong iwinyo pi gwere ki yat lageng kwidi HPV?**[kace pud pe owinyo, tite ngo ma gwer ki yat lageng kwidi HPV obedo, ci mede ki lapeny Q.26]** Kace iwinyo, ngo ma I winyo ma lube ki gwer ki yat lageng kwidi HPV ?

24. I nongo ngec ikum gwer ki yat lageng kwidi HPV ki kwene? *Mi kare ki ngat ma me gamo lapeny man ma pud peya I penyo mi niang ngec ma tut ki lapeny magi ma piny ni* [kace owaco ni peya iwinyo kit lok mo keken, Waco it gin ango ma gwer ki yat HPV obedo]

    ***Peny mi niang ma tut**: ki bot*
    - *Lamema me VHT*
    - *Lumrem onyo wadi*
    - *Lupwonye I gang kwan onyo lutic ot yot kum*
    - *Latela dini/kin gang*
    - *Radio/TV*
    - *Ikwano I gazette*
    - *I winyo iyoo ma pat?*

25. *kit kwai dano ango ma minicita me yot kum(MoH) omoko me nongo agwera me yat lageng kwidi HPV? **Peny me niang ma tut:** ki I gang kwan-joo mene? Mwaka adii? Joo ma pe kwano-joo mene? Mwakia adii? **[kace dano man pe ngeyo, wac it en kwai dull dano magi ma kwano ki ma pe gi kwano ki mwaka gi bene.]***

26. minicita me yot kum,MOH omoko ni dose adii ki kare mene ma omyero ki nong ki dose me magi wek ngat acel ace lobed ki kare me gwoko ikum nongo kwidi HPV? **[kace ngat man pe ngeyo, wac ite doc adii ma mite ki kare ma omyero onong kwede magi]**
27. kabedo mene ma pol kare ki tiyo kwede me timo/mino agwera me yat lageng kwidi HPV? **Peny me niang ma tut:** gang kwan, Ot yat, yub ikin ganag, kabedo mu kene?
28. ingeyo ngat mo onyo gurup mo ma gunongo agwera ki yat lageng kwidi HPV I kabedo ma I aa iyee? **Kace ingeyo,** kwai dano ma nining? Agwera me agiki ne otime cok coki awene I kabedo man?(peny mwaka ki dwer me agwera man ma otime ni)
29. I tamo ni agwera man obedo gin ma pire tek I kabedo ma iaa ki iyee?
30. anyaka ni dong onongo agwera me kengo kwidi HPV?**kace pud peya onongo,** pingo en pud peya ongo agwera man?**peny mi niang ma tut:** okwero yat woko, yat otum woko ki I ot yat, okweny kwan I gang kwan…

kace onongo agwera:

31. Anyaka man dong onongo agwera tyen adii?
32. Onongo agwera man ki kwene?

***Peny mi niang ma tut**: gang kwan, kilinik, ot yat, ki gang, iyoo ma pat ki magi*

33. Aduki me gwer man man anyaki ni onongo ni tye nining?
34. kit ma ineno kwede, agwera man owoto nining?
35. ii mono yom ni nyari onongo agwera ki yat lageng kwiw HPV? Waci wa pingo? En anyaka ni owaco ni inongo agwera ma owoto ni nining? Peny me niang ma pud pe onongo ki ingee nongo agwera.

***Peny mi niang ma tut**: gang kwan, ot yat, yub me kin gang,ki gang, ki kmo mukene? Pingo imito ni ki gwer ki I kabedo man*?

36. itye ki lworo onyo koko mo ma lube ki anyaka nongo agwera?
37. I ngeyo anyira ma mwaka gi rom onyo cok cok ki pa anyaka ni nini ma gi tye I gang kwan ma peya gu nongo agwera me yat lageng kwidi HPV? Kace tye, tam ango ma itwero miyo ne mi neno ni anyira ma rwatte me nong yat gu nongo yat doc aryio ducu ki I gang kwan?
38. ingeyo anyira ma mwaka gi rom onyo cok ki pa nyari ma pe gi kwano? Kit tam ango ma in I tye kwede me neno ni anyira ma mwaka opore me nongo agwera ma gitye bedo I kin ganga kany gu nongo dose aryio duc me yat lageng HPV? Peny me niang ma tut: I tamo gi twero bino ka nongo yat man I kang kwan kace ki lwongo gi? Ot yat? Yub me yot kum I kin gang? Kabedo mukene? Pingo I tamo kit man?
39. ki I tam ma megi, kabedo mene ma I neno ni opore tutwal it anyira ma pe gi kwano ma tye ikin gang kama in iaa iyee me nongo agwera me yat lageng kwidi HPV? **Peny me niang ma tut:** gang kwan, ot yat, yub me yot kum I kin gangi, kabedo mukene?
40. Pi tam ni piri keni, neno calo yoo mene ma opore me poko ngec ikum agwera ki yat lageng kwidi HPV ikin anyira ma mwaka gi rom onyo cok cok ki pa anyaka ni?(peny mi niang ma tut ikum: goyo cim it nyako man? Bot lunyodo ne? lutic yot kum? VHT? Cwalo kwena ma cek cek I cim bot lutino anyira magi? Bot lunyodo gi? Ot yat/VHT? Onyo yoo ma pat?

Miti me nongo agwera ki yat lageng kwidi HPV ki ngo ma tye ka gengo bulu ma anyira ma gitye I caro I Uganda me nongo agwera man

41. itye ki kit tam onyo lok mo ma imito medo ne ma kwako yat lageng kwidi HPV? Onyo agwera ki yat lageng kwidi HPV?

VI. Ngec ikum Agwera ki yat lageng kwidi HPV

42. lok ango ma I winyo ma ber ma lwak ma tye I kabedo I iaa ki iyee Waco ikum gwer ki yat lageng kwidi HPV? Gwere ki yat lageng kwidi HPV?
43. lworo ango ma iwinyo dano ma gitye I kabedo man gitye kwede I kum nongo agwera ki yat lageng kwidi HPV? Gwere ki yat HPV?

Peny mi niang ma tut:
-yat ma twero bedo ni petye ki kero mi kengo kwidi HPV mako anyirawa
- yat ma twero bedo ni petye ki kero mi gengo two kanca me dok onywal pa mon
-Yat man twero gengo anyirawa nywal woko
-yat man twero medo miti me butu matek tutwal bot lutino anyira
-koko mapat ki magi?

VI. Moko tam

44. itwero Waco it an lok ango ma nyari owaco it in ikum yat lageng kwidi HPV?
45. Anga ma okonyo nyari me moko tame mi nongo agwera me yat lageng kwidi HPV?

Peny me niang ma tut*: latic yot kum, VHT, lapwony I gang kwan, lurem, lanyodo, dano ma oteki, Ngat mo mapat?*

Apwoyo matek.
