# Supplementary material for: Why don’t adolescent girls in a rural Uganda district initiate or complete routine 2-dose HPV vaccine series: Perspectives of adolescent girls, their caregivers, healthcare workers, community health workers and teachers
Source: PLoS One. 2021 Jun 29;16(6):e0253735. doi: 10.1371/journal.pone.0253735 (PMC8241119; doi:10.1371/journal.pone.0253735)
Supplement: S6 File — (PDF) [file pone.0253735.s006.pdf]

Miti me nongo agwera ki yat lageng kwidi HPV ki ngo ma tye ka gengo bulu ma anyira ma gitye I caro I Uganda me nongo agwera man

S6 File. Teachers/Healthcare Providers/VHT vaccinators -- Key Informant Interview Guide Luo

I. Tuce/wiye wiye

Apwoyo bino tin! An nyinga……………………………*KET NYINGI KANY* abi bedo ka penyi lapeny ma kwako yum, kanca me dok ot nywal pa mon, yat HPV ki gwere ki yat HPV. Amito ni ibed agonya kace ma tye ka lok, ci pe ibed ki lworo mo keken kace itye ka gamo lapeny mo keken. Petye lapeny mo ma tye kakare onyo petye kakare, wamito winyo tami ikum kanca ma mako dok onywal pa mon ki gwere ki yat HPV. Lok wa ma tin twero tero dakika ma room 40-60. Abi mako dwon wa I kare me leyo tam man wek pe wakal onyo wii wa owil I kit lok mo keken ma pire tek tutwal ma in ibi leyo kwed wa. I tye ki twero pe me gamo lapeny mo keken ma miyo in pe I winyo agonya. Dok lok mo keken ma in ibi Waco it wa wabi gwoko ne I mwung dok wabi tic kede I kwan man keken. Itye ki lapeny mo? … ayela mop eke, wek wayab leyo lapeny.

II. Lok ma kwako kum in.

1. Ot yat mene ma in ki coyo in itere onyo itiyo iye?
2. Rwom tici tye ngo?
3. Itiyo pi kare ma rom mene I rwom me tic man?
4. Rwom kwani ma dit loyo ogik kwene?
5. Itye dako onyo laco?
6. Mwaka ni tye adii?

III. Yot kum kit kama inongo ngec me yot kum man iyee

7. Kong itit it an peko me yoto kum ma tye ka diyo dano I kabedo ma in iyaa iyee? ***Peny me niang ma tut*:** ma mako kum mon ki coo; awobe ki anyira
8. Lok ango me mako yot kum ma In ineno ni pire tek tutwal bot dano ma gitye I kabedo ma in iyaa iyee?
9. Yoo addii ma pii gi tegu ma dano nongo kwede ngec ma kwako yoto kum I kabedo ma in iyaa iyee?***peny mi niang ma tut:*** it coo? It mon? it awobe? It anyira?

IV. Ngec ki lok ikum kanca me dok ot nywal pa mon:

10. In tika dong iwinyo kit lok mo keken ikum kanca me dok ot nywal pa mon?
11. **Kace owinyo:** Waco kong ngo ma iwinyo ikum kanca man me dok ot nywal pa mon
    Kace pe owinyo, wac it ngat man ngo ma kanca me dok ot nywal pa mon obedo ci imede ki lappeny **Q13**.
12. Inongo ngec ikum kanca me dok ot nywal pa mon ki kewne? ***Peny mi niang ma tut***: kanya ongo lok duc ikum kanca me dok ot nywal pa mon iyee ki kit kwai lok ango ma en onongo?

- *Lamema me VHT*
- *Lumrem onyo wadi*
- *Lupwonye I gang kwan onyo lutic ot yot kum*
- *Latela dini/kin gang*
- *Radio/TV*
- *Ikwano I gazette*
- *I winyo iyoo ma pat*

Miti me nongo agwera ki yat lageng kwidi HPV ki ngo ma tye ka gengo bulu ma anyira ma gitye I caro I Uganda me nongo agwera man

13. Kit ma in ineno kwede, ngo ma in itamo I kelo kanca me dok ot nywal pa mon?
14. Joo ma gitye ii kabedo ma in iyaa Iyee gitero kanca me dok ot nywal pa mon calo gin ma piretek?
- **Kace gitero calo gin ma piretek:** pingo gitero calo gin ma piretek?
- **Kace pe gitero calo gin ma piretek:** pingo k anca me dok ot nywal pa mon pe kitero calo gin ma pire tek?
15. Lok ango ma in iwinyo ki bot dano ma gitye I kabdo ma in iaa iyee gi Waco ikum kanca me dok ot nywal pa mon pi dwei ma romo 12 angec?
16. Lok cwer cwiny onyo poto cwiny ango ma iwinyo dano ma gitye I kabedo ni Waco ikum kanca me dok ot nywal pa mon?

17. Itamo ni gin ango ma omyero kitim me gengo two kanca me dok ot nywal pa mon? ***Peny me niang matut I kum:*** gwere ki yat HPV, pime (mon ma mwaka gi oyabe 25 odok kwere malo), lok mu kene kace pe kwaco.

V. Kwidi HPV ki gwereki yat HPV:

18. I tika dong iwinyo lok mo ikum kwidi ma kelo kanca me dok ot nywal pa mon(HPV)? (kace pe owinyo, miye wiye wiye ngo ma kwid man obedo ci imede ki lapeny nama 22.)
19. Kace owinyo, knong iwac kit lok anngo ma iwinyo ikum kwid HPV? HPV obedo gin ango?
20. Inongo ngec I kum HPV man ki kwene? ***Peny mi niang ma tut:*** *ki bot*
    - *Lamema me VHT*
    - *Lumrem onyo wadi*
    - *Lupwonye I gang kwan onyo lutic ot yot kum*
    - *Latela dini/kin gang*
    - *Radio/TV*
    - *Ikwano I gazette*
    - *I winyo iyoo ma pat*
21. Dano nongo kwidi HPV nining?
    - Kobo iyoo me butu
    - Kudu kum dano
    - Leyo bongi
    - Kobo ki bot min latin ikum latin
    - Kobo ikare me medo remo I kumi
22. Kwidi HPV kelo kit kwai two ango? Mi niang ma tut:
    - Aloba loba I kum me coo onyo me mon
    - kanca mukene ma pat?
    - Kelo akwata onyo aloba kum ma pat? Onyo two ma pat?
23. Tika dong iwinyo pi gwere ki yat lageng kwidi HPV?
24. Kace iwinyo, itwero tita ngo ma iwinyo ikum gwere ki yat lagene kwidi HPV? Yat lageng kwidi HPV obedo gin ango? **Kace pe owinyo,** wac it dano man ngo ma yat lageng kwidi HPV obedo ci mede ki lapeny **Q. 26**
25. Inongo ngec ikum nongo agwera ki yat lageng kwidi HPV ki kwene? ***Peny me niang ma tut,*** *ki bot….ki kwai lok ango ma onongo ki ii kabedo acel acel*

    Inongongec ikum gwere ki yat HPV ni kikwene? ***Peny mi niang ma tut****: ki bot*
    - *Lamema me VHT*

Miti me nongo agwera ki yat lageng kwidi HPV ki ngo ma tye ka gengo bulu ma anyira ma gitye I caro I Uganda me nongo agwera man

- *Lumrem onyo wadi*
- *Lupwonye I gang kwan onyo lutic ot yot kum*
- *Latela dini/kin gang*
- *Radio/TV*
- *Ikwano I gazette*
- *I winyo iyoo ma pat*

26. Itamo ni nongo agwera ki yat HPV obedo gin ma pire tek bot joo ma gitye I kabedo ma itye iye?pingo I tamo kit meno?
27. Tika dong I bedo iyub me gwer ki yat lageng kwidi HPV I dwe ma kato 12 angec? ***Kace I bedo iye,*** *obedo I dwe mene?*
28. Minicita me yot kum kelo onyo miyo yat nining I diktrik ? **Peny me niang ma tut,** (i) kare ki kare… onyo mwaka lung? (ii) I dwe me *"child day plus"* (iii) kare ki kare ento tutwale I dwe me *"child day plus" (Kace ngat ma pe ngeyo penye kit ma minicita me yot kum oyubu kwede yub me miyo yat HPV I diktrik)*
29. Kit kwai dano mene ma minicita me yot kum omoko ni opore it gi me nongo agwera? **Peny mi niang ma tut:** kwai dano mene I gang kwan? In kin gang-kwai dano mene? Mwaka adii? **[kace ngat man pe ngeyo mwaka pa joo ma tye I gang kwan ki man tye gang pe gi kwano by minicta yee ni gu nong yat agwera man]**

30. Doc adii me yat lageng kwidi HPV ki kare mene ma opore me nongo doc magi ma minicita me yot kum(MoH) omoko ni opore it nget ma cik yee nongo agwera man wek ngat ma pe onong kwidi HPV. **[kace ngat man pe ngeyo, tite doc adii ma mite ki kare me lago kine ma MoH omoke]**
31. Pol kare gwer ki yat lageng kwidi HPV time kwene? Peny me niang ma tut ka pu owaco nyik lok magi: kang kwan, ot yat, uyb me yoto kum ikin gangi, kabedo ma pat?
32. Ingeyo ngat mo onyo joo mo ma gunongo agwera ki yat lageng kwidi HPV I kabedo ma iaa iyee?kace ingeyo, obedo kwai dano mene? Agwera me agiki otime awene I kabedo man?(peny lapeny ikum mwaka ki dwe ma otime iyee kace pe owaco)
33. Ngo ma itamo ni tye ka gengo anyira ma pe gi kwano ma mwaka gi opore me nongo agwera ki yat lageng kwidi HPV me nongo doc me acel ki me aryio me yat man? **Peny ikum jami magi k ape owaco:**
    En(ngec ma peke lworo aduki me gwer, lworo lubira, lworo ni twero meto miti me rwatte I bwutu)
    Gang(ngec ma peke, tam ma lunyodo tye kwede I kum yat me gwer man, lworo aduki me gwere ki yat man I anyim**)**
    Kin gang(lworo yat ma ki yubu ki olaya/lobo ma woko**)**
    Lok ma aa ki jami yot kum(rwec pe tye ma be ikin gangi wa pwonyo lokin gang petye ma ber, kabedo me nongo agwera boo tutwal ki kama dano bedo iyee, yat petye ma romo dano**)**
34. Ngo ma itamo ni omyero kitim wek anyira ma kare ki oromo me nongo yat ma pe gi kwano me nongo doc me acel dak me dwogo cen ka nongo doc me aryio me yat lageng kwidi HPV?
35. Ngo ma itamo ni tye ka gengo anyira ma gitye I gang kwan ma mwaka gi opore me nongo agwera ki yat lageng kwidi HPV me nongo doc me acel ki me aryio me yat man? **Peny ikum jami magi k ape owaco:**
    En(ngec ma peke lworo aduki me gwer, lworo lubira, lworo ni twero meto miti me rwatte I bwutu)

Miti me nongo agwera ki yat lageng kwidi HPV ki ngo ma tye ka gengo bulu ma anyira ma gitye I caro I Uganda me nongo agwera man

Gang(ngec ma peke, tam ma lunyodo tye kwede I kum yat me gwer man, lworo aduki me gwere ki yat man I anyim**)**
Kin gang(lworo yat ma ki yubu ki olaya/lobo ma woko**)**
Lok ma aa ki jami yot kum(rwec pe tye ma be ikin gangi wa pwonyo lokin gang petye ma ber, kabedo me nongo agwera boo tutwal ki kama dano bedo iyee, yat petye ma romo dano**)**

36. Ngo ma itamo ni omyero kitim me meto miti it anyira ma kare ki oromo me nongo yat ma gitye I gang kwan me nongo doc me acel? dak me dwogo cen ka nongo doc me aryio me yat lageng kwidi HPV?
37. Ngo ma itamo ni omyero kitim me meto miti it anyira ma gi bedo gang pe gi kwano ma kare ki oromo me nongo yat ma pe gi kwano me nongo doc me acel ?dak me dwogo cen ka nongo doc me aryio me yat lageng kwidi HPV?
38. Lok ango maber ma iwinyo dano ma gitye I kabedo ma iaa iye Waco ikum agwera ki yat lageng kwidi HPV?
39. Lwoko onyo koko ango ma iwinyo dano tye kwede ikum nongo gwer ki yat lageng kwidi HPV? **Peny Ikum:** lworo dugu lalur, meto miti me rwatte I buttu, aduki me gwer, lok mukene ma pat?
40. Tika dong iwinyo peko me yot kum mo ma obino calo awoki me agwera ki yat lageng kwidi HPV? Kace winyo, itwero waci wa peko man ki ngo ma otime?
41. Ikit na in itamo kwede, itamo ni (i) kanca me dog ot nywal pa mon(ii) yat agwera me lageng Kwidi HPV (iii) Nongo agwera ki yat lageng kwidi HPV tye gin ma pire tek I kabedo ma I aa iye? Pingo I Waco kit man?

VI. Meto ngec ikum gwer ki yat me lageng kwidi HPV

42. ngo ma in itamo ni obedo beco pa nongo gwer ki yat lageng HPV?
43. yoo mene ma I neno calo ber loyo me pwonyo anyira ki lunyodo gi ikum ber pa gwere ki yat lageng kwidi HPV?
44. itamo yoo mene ma ki twero tic kwede me miyo miti it anyira ma tye I kabedo I aa iyee mi neno ni gu nongo agwera me gengo kwidi HPV? Lunyodo/ lagwok gi? Lutic yot kum/VHT? Lupwonye?
Peny mi niang ma tut kace pu owaco jami magi:
    - mwono gin akwana ma pol it ot yat, gangi kwan, gang lega.
    - kace ngat mo ma ki woro matek oloko I gang lega, gang kwn, radion onyo kacoke me kiyubu I kin gang.
    - cwallo lok akwana ma cek cek I cim bot lanyodo onyo lagwok latin? Calo lapo wic I cim
    - pwonyo lanyodo onyo lagwoko latin I kum gwere me gengo kwidi HPV ki cwalo lok akwna ma cek cek I cim?
    - cwalo lok angeya I email onyo cim bot lutic yot kom ki VHT ma gwero dano?
    - yubu pwonye matino tino me poko ngec ikum ka kwene ma ki twero nongo agwera iyee.
    - kace latic yot kum oloko gi anyira wa ki luyodo pa anyira ma kare gi oromo onyo twero gwere ki I gang kwan onyo I yub kacoke ma kiyubu I kin gang.

45. Ngo ma itam ni ki twero timo ne iyoo ma pat ki kit ma ki tye katimo ne kum kare ni ma lube ki(i) ma pud me ki yabo gwer ki (ii) ingee gwer ki yat me gengo kwidi HPV me miyo miti it anyira weny ma twero nongo ma **gi tye I gang kwan** me nongo agwera? ***Peny me niang ma dwong I kom:***

- Pwonyo lutic yoto kum ki lupwonye I gang kwan, pwonye ki.
- Yubu kit me kube ikin gangi kwan ki anyira ma kare ki oromo gwer ki dong joo ma gwero gwer.
- Yubu kit me poko yat agwera me gengo kwidi HPV .ma
- Yubu kit me dingo lutic yot kum ma gi gwera dano me tero gi kanya gwer tye ka time iyee.

Miti me nongo agwera ki yat lageng kwidi HPV ki ngo ma tye ka gengo bulu ma anyira ma gitye I caro I Uganda me nongo agwera man

- Medo rwom ma lunyodo/ lugwok nyako me bedo tye I kare me gwero lutino gi.
- Meto tic pa lapwony in ibedo iyub me gwer me gengo kwiti HPV.
- Culu mucara pa lutic labongo galle.
- Tam ma pat ki magi?

46. Ngo ma itam ni ki twero timo ne iyoo ma pat ki kit ma ki tye katimo ne kum kare ni ma pud/ ingee gwer ki yat me gengo kwidi HPV me miyo miti it **anyira weny ma peke I gangi kwan** magi bedo I kin gang kany me nongo agwera?

Peny me niang ma dwong I kom:

- Pwonyo lutic yoto kum ki lupwonye I gang kwan, pwonye ki.
- Yubu kit me kube ikin gangi kwan ki anyira ma kare ki oromo gwer ki dong joo ma gwero gwer.
- Yubu kit me poko yat agwera me gengo kwidi HPV .ma
- Yubu kit me dingo lutic yot kum ma gi gwera dano me tero gi kanya gwer tye ka time iyee.
- Medo rwom ma lunyodo/ lugwok nyako me bedo tye I kare me gwero lutino gi.
- Meto tic pa lapwony in ibedo iyub me gwer me gengo kwiti HPV.
- Culu mucara pa lutic labongo galle.

47. ngo ma in itamo ni gengo nyako ma onongo gwer me acel(dose me acel) me nongo dose/gwer me aryio me yat lageng kwidi HPV? ***Peny me niang ma tut I kum jami magi:***

- Lanyodo/lagwok latin pe miyo twero me gwero latin
- Latin anyaka gwero woko ni ki gwere
- Pe tye kit niang latic yot kom mo keken ma ogero gwer I buk me gwer ki me yat lagen HPV.
- Bedo peke I gang kwan I nino gwer
- Kobo/loko gang kwan
- Kwero kwan
- Yat tum woko ki I ot yat
- Lworo lubira
- Lworo aduki pa yat
- Pi tien lok ma pat?

48. ngo ma in I tamo ni omyero ki tim wek anyira ma onongo gwer me cel mi dwoko cen kanongo gwer me aryio me yat lagen kwidi HPV
49. I tye ki kit lok mo keken ikum gwere ki yat lagen HPV?

Apwoyo matek.
